# Cell-Selective Adeno-Associated Virus-Mediated *SCN1A* Gene Regulation Therapy Rescues Mortality and Seizure Phenotypes in a Dravet Syndrome Mouse Model and Is Well Tolerated in Nonhuman Primates

**DOI:** 10.1089/hum.2022.037

**Published:** 2022-06-10

**Authors:** Annie Tanenhaus, Timothy Stowe, Andrew Young, John McLaughlin, Rangoli Aeran, I. Winnie Lin, Jianmin Li, Raghavendra Hosur, Ming Chen, Jennifer Leedy, Tiffany Chou, Sirika Pillay, Maria Candida Vila, Jennifer A. Kearney, Martin Moorhead, Archana Belle, Stephanie Tagliatela

**Affiliations:** ^1^ Encoded Therapeutics, Inc., South San Francisco, California, USA.; ^2^ Department of Pharmacology, Northwestern University Feinberg School of Medicine, Chicago, Illinois, USA.

**Keywords:** SCN1A, Dravet syndrome, encephalopathy, channelopathy, gene regulation therapy, preclinical models

## Abstract

Dravet syndrome (DS) is a developmental and epileptic encephalopathy caused by monoallelic loss-of-function variants in the *SCN1A* gene. *SCN1A* encodes for the alpha subunit of the voltage-gated type I sodium channel (Na_V_1.1), the primary voltage-gated sodium channel responsible for generation of action potentials in GABAergic inhibitory interneurons. In these studies, we tested the efficacy of an adeno-associated virus serotype 9 (AAV9) *SCN1A* gene regulation therapy, AAV9-RE^GABA^-eTF^*SCN1A*^, designed to target transgene expression to GABAergic inhibitory neurons and reduce off-target expression within excitatory cells, in the *Scn1a*^+/−^ mouse model of DS. Biodistribution and preliminary safety were evaluated in nonhuman primates (NHPs). AAV9-RE^GABA^-eTF^*SCN1A*^ was engineered to upregulate *SCN1A* expression levels within GABAergic inhibitory interneurons to correct the underlying haploinsufficiency and circuit dysfunction. A single bilateral intracerebroventricular (ICV) injection of AAV9-RE^GABA^-eTF^*SCN1A*^ in *Scn1a*^+/−^ postnatal day 1 mice led to increased *SCN1A* mRNA transcripts, specifically within GABAergic inhibitory interneurons, and Na_V_1.1 protein levels in the brain. This was associated with a significant decrease in the occurrence of spontaneous and hyperthermia-induced seizures, and prolonged survival for over a year. In NHPs, delivery of AAV9-RE^GABA^-eTF^*SCN1A*^ by unilateral ICV injection led to widespread vector biodistribution and transgene expression throughout the brain, including key structures involved in epilepsy and cognitive behaviors, such as hippocampus and cortex. AAV9-RE^GABA^-eTF^*SCN1A*^ was well tolerated, with no adverse events during administration, no detectable changes in clinical observations, no adverse findings in histopathology, and no dorsal root ganglion-related toxicity. Our results support the clinical development of AAV9-RE^GABA^-eTF^*SCN1A*^ (ETX101) as an effective and targeted disease-modifying approach to SCN1A^+^ DS.

## INTRODUCTION

Dravet syndrome (DS) is a severe, early-onset developmental and epileptic encephalopathy and channelopathy that significantly impacts affected children and their families. DS is manifested by frequent prolonged seizures, status epilepticus events, significant cognitive delays, sleep abnormalities, motor impairment, and profound behavioral difficulties.^1–6^ Up to 20% of DS patients die before adulthood, frequently due to sudden unexpected death in epilepsy (SUDEP).^7,8^ DS is a rare disease with an estimated prevalence of 1 in 15,500 live births.^9^

Over 85% of DS cases are caused by heterozygous loss-of-function variants in a single copy of the *SCN1A* gene, encoding the alpha subunit of the voltage-gated type I sodium channel (Na_V_1.1).^10^ Na_V_1.1 is predominantly expressed within the axon initial segment of GABAergic inhibitory interneurons, where it generates and propagates action potentials.^11^ Genetic reduction of Na_V_1.1 substantially reduces the frequency and amplitude of action potentials generated by GABAergic inhibitory interneurons, thereby impairing their inhibitory function, which is dependent on high-frequency firing.^12–14^

Multiple lines of evidence have established impaired excitability of GABAergic inhibitory interneurons as the central driver of key DS phenotypes, including seizures, mortality, and cognitive deficits.^12,13,15–21^ Specific deletion of *SCN1A* within forebrain GABAergic inhibitory interneurons is sufficient to recapitulate sensitivity to hyperthermia-induced seizures (HTS), premature death, as well as behavioral and cognitive impairments observed in global *SCN1A* knockouts.^13,15^ Furthermore, enhancement of GABAergic activity with the allosteric GABA-A receptor agonist, clonazepam, rescues abnormal social behaviors and learning and memory deficits in DS mice.^13^ Taken together, this evidence suggests that a cell-specific genetic approach aimed at restoring expression levels and function of *SCN1A* within GABAergic inhibitory interneurons would have the potential to address the underlying cellular and molecular etiology of SCN1A^+^ DS while minimizing potential off-target effects of Na_V_1.1 expression.

Gene replacement therapy using recombinant, nonreplicating adeno-associated virus (AAV)-based vectors has demonstrated transformative clinical benefits for the treatment of severe monogenic central nervous system (CNS) disorders.^22^ However, as the *SCN1A* gene (cDNA ∼6 kb) exceeds the packaging capacity of AAV (4.7 kb DNA),^23^ AAV-mediated gene replacement therapy for SCN1A^+^ DS is not a viable approach. Therefore, we developed AAV9-RE^GABA^-eTF^*SCN1A*^ (ETX101), an investigational AAV-mediated *SCN1A* gene regulation therapy candidate, which expresses an engineered transcription factor (eTF^*SCN1A*^) designed to upregulate the *SCN1A* gene from the endogenous genome. A cell-selective regulatory element (RE^GABA^) was incorporated to target transgene expression specifically to GABAergic inhibitory interneurons.

In these studies, we utilize two previously described DS mouse models,^11,24,25^ and demonstrate that AAV-mediated upregulation of *SCN1A* with AAV9-RE^GABA^-eTF^*SCN1A*^ increases *SCN1A* transcripts specifically within GABAergic inhibitory interneurons, prolongs survival, and reduces spontaneous seizures and HTS. In nonhuman primates (NHPs), a one-time unilateral intracerebroventricular (ICV) injection of AAV9-RE^GABA^-eTF^*SCN1A*^ led to widespread vector biodistribution and robust transgene expression throughout the brain, including forebrain, midbrain, and particularly the cortex and hippocampus, two key structures involved in epilepsy and cognitive symptoms in DS. ICV-administered doses of AAV9-RE^GABA^-eTF^*SCN1A*^ at up to 8E13 vector genomes (vg) per animal were well tolerated, with no detectable changes in clinical observations, no adverse findings in histopathology, and no dorsal root ganglion (DRG)-related toxicity. Our results support the clinical development of AAV9-RE^GABA^-eTF^*SCN1A*^ gene regulation therapy for the treatment of SCN1A^+^ DS.

## EXPERIMENTAL PROCEDURES

### Vector design: eTF^*SCN1A*^ and RE^GABA^

**Figure 1.**
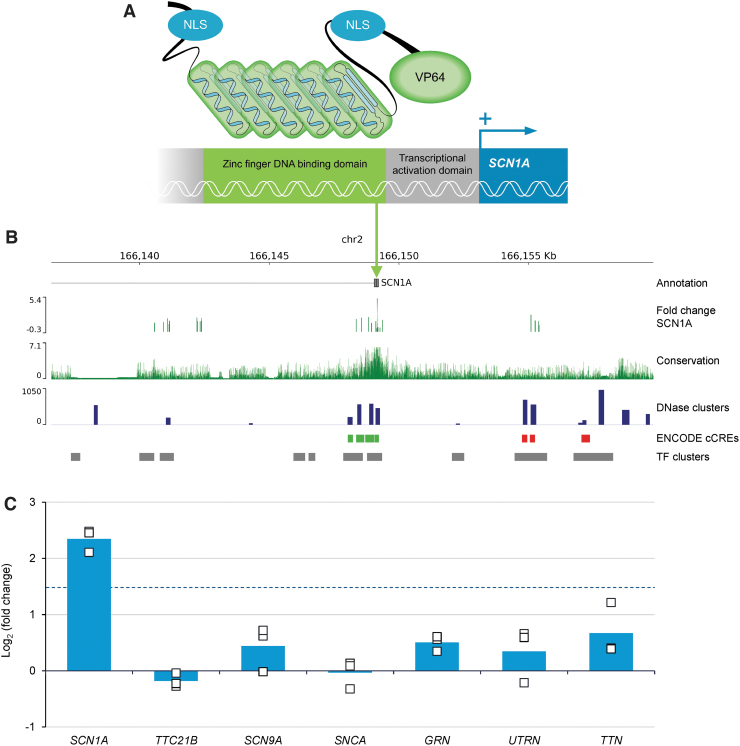
Structure of eTF^*SCN1A*^ and its targeted activity at a unique and highly conserved 18-bp noncoding DNA sequence for gene-specific upregulation of *SCN1A*. **(A)** Schematic of eTF^*SCN1A*^ structure. eTF^*SCN1A*^ is composed of a polydactyl zinc finger DNA-binding domain, a short linker sequence, and a C-terminal VP64 transcriptional activation domain.^26,28^ Each of the six zinc finger domains interacts with a specific triplet nucleotide, conferring specific interaction with an 18-bp target sequence. NLS domains derived from simian vacuolating virus (SV40) and nucleoplasmin are included at the N-terminal and linker domains. **(B)** Genomic features of eTF^*SCN1A*^ target site. Human genome track at the *SCN1A* locus. Tracks indicate human *SCN1A* gene annotation, chromosome 2 coordinates (hg38 genome assembly). FC SCN1A track shows target vector candidate screening data in HEK293cells. Mean Log2-FCs in SCN1A expression following transient transfection of eTF^*SCN1A*^ candidates targeted against different genomic targets are plotted by target coordinates. The eTF^*SCN1A*^ 18-bp target sequence, which induced the strongest upregulation of SCN1A within the screening window, is indicated by the *green arrow*. Sequence conservation track is indicated by 100-species base-wise conservation score (PhyloP).^29^ Regulatory element feature tracks are indicated (UCSC genome browser, ENCODE consortia^31,32^). DNase cluster track indicates DNase I hypersensitivity cluster score; ENCODE cCREs track marks candidate cis-regulatory element regions (*green*: PLS, *red*: dELS); TF clusters track marks transcription factor binding peak clusters. **(C)** eTF^*SCN1A*^ upregulates *SCN1A* in a gene-specific manner *in vitro*. Change in endogenous gene expression in HEK293 cells is reported in the eTF^*SCN1A*^ transfected condition relative to control condition for each replicate. Bars represent mean Log2-FC for *n* = 3 biological replicates (*n* = 2 for PAPBC1), *dots* indicate individual replicate measurements. *Dotted line* indicates Log2FC = 1.5. bp, base-pair; cCREs, candidate cis-regulatory elements; dELS, distal enhancer-like signature; FC, fold change; NLS, nuclear localization signal; PLS, promoter-like signature; TF, transcription factor.

AAV9-RE^GABA^-eTF^*SCN1A*^ was developed through interrogation of the *SCN1A* gene and human genetic sequences surrounding the *GAD1* gene, expressed specifically in GABAergic interneurons. The selectivity of the backbone of AAV9-RE^GABA^-eTF^*SCN1A*^ was determined by generating vectors that expressed visualization markers (enhanced green fluorescent protein [EGFP]). The design of eTF^SCN1A^ was based on the Barbas zinc finger design principles,^26,27^ whereby 6-finger domains targeting defined 18 base-pair (bp) target sequences can confer genome-wide specificity.^28^ A VP64 transcriptional activation domain, as well as nuclear localization signals at the N-terminal and linker regions were included to facilitate transcriptional upregulation (Fig. 1A).^27^

We designed several candidate target sequences based on conservation (PhyloP)^29,30^ and genomic regulatory markers surrounding the distal *SCN1A* promoter (NM_001202435), including species sequence conservation (Fig. 1B),^29^ accessibility data (DNase hypersensitivity), and transcription factor binding site enrichment (chromatin immunoprecipitation sequencing [ChIP-seq] clusters) (Fig. 1B).^31,32^ Based on these features, candidate sequences were screened empirically for efficacy in upregulating *SCN1A* expression (see Fig. 1B, “fold change SCN1A” track). *SCN1A* upregulation and genetic selectivity were evaluated by reverse transcription–quantitative polymerase chain reaction (RT-qPCR) *in vitro* through transient transfection studies.

In brief, adherent HEK293 cells (293AAV; Cell Biolabs) were cultured and transfected (FuGENE HD; Promega) with either an eTF^*SCN1A*^-expressing plasmid construct or an EGFP-expressing control 24 h postplating. RNA was isolated (RNeasy Mini kit; Qiagen) 48 h post-transfection, DNase treated, and reverse transcribed using OligoDT primers (Superscript IV; Invitrogen; Supplementary Table S1). cDNA samples were analyzed by qPCR and analysis of relative expression was performed using the ΔΔCt method. Referenced ENCODE datasets were accessed from the ENCODE portal (https://www.encodeproject.org/)^33^ or UCSD Genome Browser^34^ using the Table Browser tool (https://www.genome.ucsc.edu).^35^ Accession numbers for underlying data are EH38E1393970, GSE29692, GSE32970, and GSE26386.

### AAV vector production

Replication-incompetent, recombinant AAV9 particles were produced in HEK293 cells via transient triple cotransfection: a transgene-containing plasmid, a packaging plasmid for Rep and Cap genes, and a plasmid containing adenoviral helper genes. Following transfection, cells were harvested, lysed to release viral particles, and treated with Benzonase. For mouse studies, AAV9 viral vector produced in the adherent HEK293 system was purified by iodixanol gradient followed by buffer exchange (video electroencephalography [EEG] studies), or generated by Vector Biolabs (Malvern, PA) using an adherent HEK293 system and purification by CsCl centrifugation (all other mouse studies). Vector was formulated in phosphate-buffered saline (PBS) and stored at −80°C. AAV vector titer for each production was originally measured by qPCR method and subsequently retentates were retitered using a qualified digital droplet polymerase chain reaction (ddPCR) assay (reported values).

For the primate study, AAV9 vector was produced at larger scale for both adherent and suspension HEK293 production platforms. Viral vector produced in the adherent platform was purified by iodixanol gradient and followed by ion exchange chromatography. AAV vector produced in the suspension platform was purified using chromatography-based methods. Vector was formulated in PBS with 0.001% pluronic and titer was determined using a qualified ddPCR method.

### Animals

Mouse studies were conducted at Encoded Therapeutics, Inc. and performed under protocols approved by the Institutional Animal Care and Use Committee (IACUC) in accordance with the National Institutes of Health *Guide for the Care and Use of Laboratory Animals*. Heterozygous *Scn1a*^+/−^ breeders were generously provided by Dr. Jennifer Kearney at Northwestern University (Chicago, IL). F1 C57:129S hybrid *Scn1a*^+/−^ mice were crossed to 129S6/SvEvTac *Scn1a*^+/−^ mice to generate F1 offspring. *Scn1a^R1407X/+^* mice (R1407X) were obtained from Riken University. Heterozygous animals were generated at Charles River Laboratories by fertilizing ova from C57BL/6N mice with sperm from *Scn1a^R1407X/+^* mice followed by transplantation of fertilized embryo into CD-1 surrogates. Genotyping was performed by automated RT-PCR at TransnetYX, Inc. [see Supplementary Materials for primer sequences].

All mice were maintained on a 12:12-h light:dark cycle and had *ad libitum* access to food and water throughout the experiments. Mice were euthanized using CO_2_ followed by cervical dislocation. For molecular analysis, cortical brain tissues were collected, flash frozen, and stored at −80°C. For immunohistochemistry (IHC), brains were collected and preserved in 10% neutral buffered formalin for 24 h then switched to 70% ethanol and stored at 4–8°C until histopathologic processing.

NHP studies were conducted using AAV9-seronegative juvenile cynomolgus macaques (*Macaca fascicularis*) at Charles River (Mattawan, MI), and were performed according to local health authority guidelines. Sera were screened for preexisting AAV9 neutralizing antibodies and animals with titers <1:5 were selected. Animals were group-housed in acclimatized holding rooms with water *ad libitum*. Animals were given meals of balanced composition and additional food was offered to provide environmental enrichment. At the end of the study, euthanasia procedures were conducted in accordance with CR-M protocols and standard operating procedures, and brain tissues were collected.

The whole brain from rostral to caudal was cut into coronal 5-mm thick slabs, and interleaving slabs were either fixed for histology in 4% paraformaldehyde for 24–48 h then transferred and stored in 70% ethanol until processing, or 8-mm punches were taken across nine brain regions in RNAlater™ or flash-frozen for DNA/RNA/protein analysis. The spinal cord was removed, and the cervical C5, thoracic T4 and T11, and lumbar level L3 of spinal cord and associated DRGs were collected. Tissue samples were shipped under appropriate conditions from Charles River to Encoded Therapeutics, Inc.

### ICV injections

At postnatal day (PND)1, mice were anesthetized on ice for ∼3 min. Three microliters of viral suspension or vehicle control were administered into each hemisphere using an insulin syringe. Lambda and bregma skull sutures were referenced to determine injection site ∼1 mm lateral, 1 mm rostral, respectively. The needle was lowered to an approximate depth of 1.5 mm ventral for injection. After injection, mice were placed in a warming pad and returned to the mother in the cage.

For unilateral ICV administration in juvenile cynomolgus macaques, presurgical MRI was performed to establish brain ventricle coordinates within the left or right hemisphere. A small burr hole was made through the frontal bone and a spinal needle (22 gauge) was introduced. The correct needle placement was verified by injecting up to 0.05 mL of contrast agent (Omnipaque™) under real-time fluoroscopy examination. Then, a controlled volume of vehicle or test article of 2.0 mL was injected at a rate of 0.1 mL/min.

### Immunohistochemistry

IHC was performed on serial section slides in two independent multiplexed batches. The first batch followed the sequence mouse anticalcium/calmodulin-dependent protein kinase II alpha (MA1-048; Invitrogen), rabbit antisomatostatin (T-4102; Peninsula Laboratories), rabbit anti-GFP (ab290; Abcam), and mouse anti-PV (PV235; Swant). The second batch followed the sequence, mouse anti-glutamate decarboxylase (GAD67, ab26116; Abcam), mouse anti-fox-3 (NeuN, MAB 377; Millipore), and rabbit anti-GFP. After primary and secondary antibody incubation, slides were stained with DAPI. Whole slide images were captured at 20 × using an Akoya Biosciences Polaris instrument. Image exposures for each fluorochrome were constant for all slides. Detailed image analysis is described in the Supplementary Materials.

### NHP histopathology

At necropsy, whole brain and spinal cord with associated DRGs were collected and processed for formalin-fixed paraffin embedding. Tissues were sectioned at 5 μm and stained with hematoxylin and eosin on 16–20 slides per animal. Macroscopic and microscopic morphologic observations and evaluations were obtained from three independent board-certified veterinary pathologists.

### Quantification of vector copy number by ddPCR

DNA was isolated from tissue using AllPrep DNA/RNA Mini Kit (Qiagen) according to the manufacturer's instructions. TaqMan primers were directed against regions of transgene (eTF^*SCN1A*^) sequence (eTF^*SCN1A*^ forward primer 5′-GAATGTGGGAAATCATTCAGTCGC-3′, eTF^*SCN1A*^ reverse primer 5′-GCAAGTTATCCTCTCGTGAGAAGG-3′, eTF^*SCN1A*^ probe 5′-GCGACAACCTGGTGAGACATCAACGCACC-3′) and mouse *Tfrc* (*MmTfrc*) gene or monkey *Albumin* (Mf*Alb*) gene as an internal control for normalizing genomic DNA content for mouse tissues. Droplets were generated and templates were amplified using automated droplet generator and thermo cycler (Bio-Rad). After the PCR step, the plate was read by QX2000 Droplet Reader to quantify vector copy number (VCN) levels in tissues. VCN per microgram of DNA was converted to copy number per diploid genome.

### Quantification of RNA expression by RT-ddPCR

RNA was isolated using AllPrep DNA/RNA Mini Kit (Qiagen) according to the manufacturer's instructions. TaqMan primers were directed against regions of transgene (eTF^*SCN1A*^) sequence and mouse *GUSB* (Mm*GUSB*) gene or monkey *ARFGAP2* (Mf*ARFGAP2*) gene as an internal control for normalizing genomic DNA content for mouse tissues (Supplementary Table S2). The no-RT reaction served as negative control for each sample. Droplets were generated and templates were amplified using automated droplet generator and thermo cycler (Bio-Rad). Gene expression copies per microgram RNA was calculated based on the output of QX2000 Droplet Reader (copies per microliter ddPCR reaction) multiplied by the total reaction volume and divided by the RNA input.

### Single-nucleus RNA sequencing

Brain tissue was homogenized as previously described.^36^ The nuclei preparations were stained with DAPI, and 100,000 DAPI-positive events were sorted using a BD FACS Aria II cell sorter (UCSF Gladstone Flow Cytometry Core Facility). Single-nucleus RNAseq (snRNAseq) was performed using the Chromium Single Cell 3′ v3 kit (10 × Genomics), according to manufacturer's instructions. An enrichment PCR was utilized to specifically amplify eTF cDNA, thereby improving the sensitivity of detection of eTF^*SCN1A*^ within the cDNA pool. The resulting cDNA libraries and enrichment PCR products underwent next-generation sequencing using a NextSeq 500 (Illumina). CellRanger (v5.0) was used to align reads and generate UMI (Unique Molecular Identifier) counts for each gene.

These counts were used for dimensionality reduction (Seurat 4.0 [https://satijalab.org/seurat/] and ZINB-WaVE^37^) and clustering using the Louvain algorithm (https://pypi.org/project/louvain/, version 0.6.1) to define neuronal subpopulations by their genetic signatures. Cells with ≥1 UMI count for the eTF^*SCN1A*^ sequence were considered “infected,” whereas cells with no UMI count for the eTF^*SCN1A*^ sequence were considered “uninfected.”

### Quantification of membrane-associated Na_V_1.1 protein by Meso Scale Discovery electrochemiluminescence-based sandwich immunoassay

Mouse forebrain and midbrain tissues were homogenized using a Qiagen TissueLyser II, according to manufacturer's instructions, and plasma membranes isolated by a series of centrifugation steps. Total protein concentration was determined using the Pierce™ BCA Protein Assay Kit (Thermo Fisher Scientific, Inc.) and total protein 1 mg/mL was loaded onto a Meso Scale Discovery (MSD) plate for measurement via an electrochemiluminescence-based sandwich immunoassay. Membrane-associated Na_V_1.1 was detected with a rabbit anti-Na_V_1.1 pAb against the C-terminus of mouse Na_V_1.1 as capture antibody and a mouse mAb against the same molecular region as detection. For Na_V_1.1 quantification, a recombinant mouse Na_V_1.1 C-terminus protein fragment was used as reference standard. The level of membrane-associated Na_V_1.1 in the sample was normalized by total protein loaded in each well and reported as percentage of that in wild-type (WT) mice.

### HTS assay

Mice were placed in a TCAT 2DF (Physitemp) box for 15 min before assay initiation. A flexible rectal probe (RET-4; Physitemp) was inserted in the mice and secured with medical tape. Mice were placed in a 4-L beaker under a heat lamp and body temperature was increased by ∼0.5°C every 2 min until the onset of the first tonic-clonic seizure, defined by uncontrolled movements accompanied by loss of posture, or until 43.5°C was reached (as described previously^38^). Seizure events, defined as tonic-clonic seizures with loss of posture, were recorded by an experimenter blinded to treatment group and genotype. The experiment was terminated when the first tonic-clonic seizure occurred or when the mouse reached 43.5°C for 2 min.

### Video-EEG seizure analysis

*Scn1a^+/^*^−^ and WT mice were implanted with three electrodes on PND21: one each in the frontal cortex, parietal cortex, and hippocampal regions. Video-EEG recordings were performed for nine consecutive days using the Pinnacle Technology 8206 data conditioning and acquisition system (Pinnacle Technology, Inc.). Electrographic seizure events were identified with automated spike detection. Spike trains consisting of a clustering of ≥10 spikes with amplitude >3 × basal EEG background activity were considered electrographic seizure events and were manually confirmed by accompanying video recordings.

Behavioral scoring for all electrographic seizure events was evaluated based on the following criteria: Nonbehavioral seizure: no observed change in behavior; Mild seizure: mild movements with or without head twitching; Convulsive seizure: tonic-clonic seizure with or without loss of posture. Electrographic seizure events that demonstrated spiking activity across all three EEG channels were considered generalized seizure events. All assessments were performed by experimentalists blinded to treatment.

### Statistical analyses

For *in vitro* RNA expression analysis, RT-qPCR and RT-ddPCR analyses were performed in triplicate biological samples, and one-sample *z*-tests were used to calculate if the mean Log2-fold change (Log2FC) was significantly different from zero. Differences in expression for each gene were considered significant if they passed differential expression thresholds of mean Log2FC ≥1.5 or mean Log2FC ≤ −1.5. Log-rank test was performed on HTS assay and survival data. Unpaired *t*-tests were used to compare EEG seizure frequency between groups and for IHC colocalization analysis. Chi-squared test was used to compare EEG seizure severity. *p*-Values are denoted as: *p < 0.05; **p < 0.01; ***p < 0.001; ns, not significant, *p* ≥ 0.05.

## RESULTS

### eTF^*SCN1A*^ targets a unique and highly conserved 18-bp noncoding DNA sequence and upregulates *SCN1A* in a gene-specific manner

To achieve selective upregulation of *SCN1A* expression in GABAergic inhibitory interneurons, a GABAergic cell-selective regulatory element (RE^GABA^) and a transgene expressing an engineered transcription factor targeted to the *SCN1A* gene (eTF^*SCN1A*^) were incorporated into AAV9 vector for transgene delivery, referred to as AAV9-RE^GABA^-eTF^*SCN1A*^. eTF^*SCN1A*^ comprises a synthetic DNA-binding zinc-finger protein fused to the transcriptional activator, VP64 (Fig. 1A),^26,28^ and was designed to selectively bind a conserved regulatory region upstream of the *SCN1A* transcription start site (NM_001202435) (see Experimental Procedures section). Within this promoter region, candidate target sequences were screened *in vitro* by transient transfection, and an 18-bp sequence element was identified that produced robust upregulation of *SCN1A* exceeding threshold criteria (Log2FC >1.5, *p* < 0.05) (Fig. 1B).

This target binding site corresponds to a peak in multispecies sequence conservation, across human, mouse, and NHP genomes (Fig. 1B), allowing the use of preclinical models to assess the safety and efficacy of transcriptional activation of *SCN1A*.

Next, we evaluated specificity of the eTF^*SCN1A*^ transgene *in vitro* by transient transfection. HEK293 cells were transfected with plasmids expressing either the eTF^*SCN1A*^ transgene or EGFP under the control of a strong constitutive promoter element. While transfection with eTF^*SCN1A*^ resulted in upregulation of SCN1A transcript, no significant upregulation was observed in a panel of six off-target genes, including the two nearest neighboring genes to *SCN1A* (*TTC21B, SCN9A*) and four additional control genes (*SNCA*, *GRN*, *UTRN*, *TTN*) (Fig. 1C). These results indicate that upregulation by eTF^*SCN1A*^ is selectively targeted to the *SCN1A* gene, and not a general feature of transcriptional activator protein overexpression.

To evaluate potential genomic off-target binding sites, BLASTn (NCBI) analysis and target annotation were performed against human genome on the 18-bp target sequence. Importantly, this sequence is unique within the human genome, with few predicted off-target binding sites. None of the identified homologous binding sites overlapped with identified promoter or enhancer regions (Supplementary Table S3).

### RE^GABA^ selectively targets GABAergic interneurons *in vivo*

**Figure 2.**
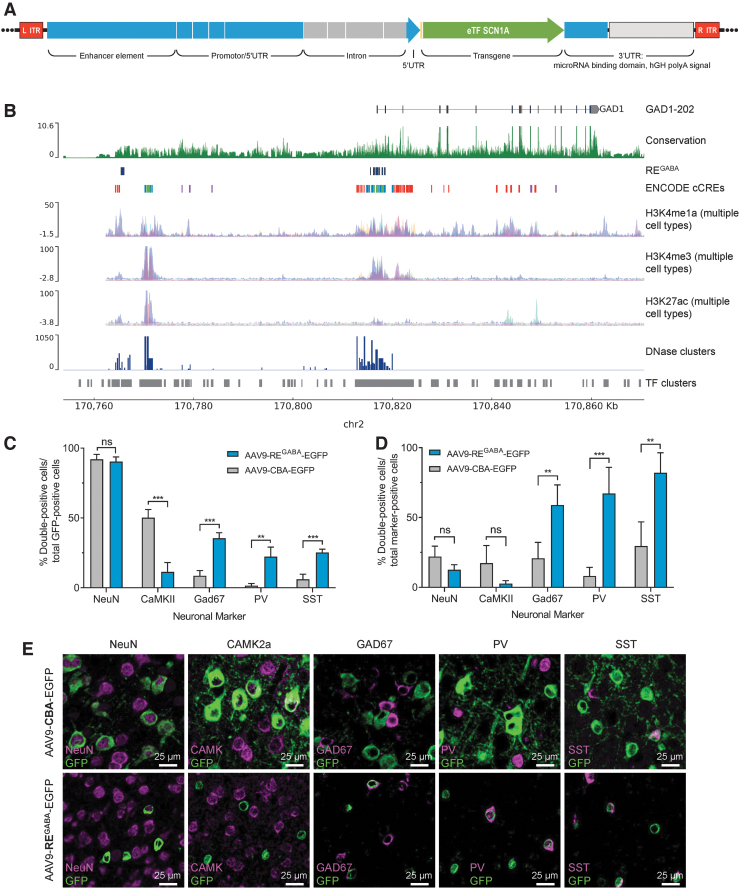
The RE^GABA^ regulatory element in AAV9-RE^GABA^-eTF^*SCN1A*^ selectively targets GABAergic interneurons *in vivo*. **(A)** RE^GABA^ construct design. RE^GABA^ comprised upstream and 5′UTR sequence components, and a downstream 3′UTR sequence. The upstream regulatory sequence is derived from human genomic sequence elements: an enhancer element located ∼50 kb upstream of the *GAD1* gene locus, and proximal promoter and 5′UTR element sequence derived from the human *GAD1 gene* locus. The promoter is composed of genomic sequence segments in the proximal promoter and 5′UTR of the *hGAD1* gene. To enhance gene expression^40^ and to capture additional regulatory features derived from intronic elements, this promoter also incorporates a truncated intron derived from the first intron of the *GAD1* gene, which includes sequences flanking the splice donor and acceptor sites, and internal intronic sequences that overlap high-conservation predicted regulatory sequences. The 3′UTR element includes a collection of eight cognate target motifs derived from excitatory neuron-enriched miRNAs miR128 and mir221,^36,39^ and a hGH-pA. **(B)** Genomic features of RE^GABA^ source sequence. Human genome track at the human *GAD1* locus. Tracks indicate *GAD1* gene annotation, chromosome 2 coordinates (hg38 genome assembly). Sequence conservation (PhyloP^29,30^), and ENCODE regulatory element feature tracks are indicated, as well as additional composite epigenetic marker tracks for histone markers H3K4me1, H3K4me3, and H3K27ac, which can signal active enhancer and promoter regions (UCSC genome browser, ENCODE consortia). **(C–E)** Expression of EGFP *in vivo* 27 days post-ICV injection of AAV9-EGFP vectors driven by CBA or RE^GABA^ promoters in PND1 mice. Animals (*n* = 4/group) were administered 2.0E10 vg per animal of AAV-CBA-EGFP or AAV-RE^GABA^-EGFP on PND1 by bilateral ICV injection. On PND28, brain sections were analyzed by IHC for the presence of GFP and neuron-specific markers. Quantitation of colocalization between neuron-specific markers^a^ and EGFP: **(C)** among GFP-positive cells and **(D)** compared to the total number of neuron-marker positive cells; **p < 0.01; ***p < 0.001; ns, *p* ≥ 0.05 (unpaired *t*-tests, two-stage step-up FDR = 1%; data shown are means and error bars represent SD). **(E)** Representative images demonstrating colocalization of EGFP driven by each vector with each of the neuronal markers evaluated. ^a^Neuron-specific markers: NeuN (neurons); CAMK2a (glutamatergic neurons); GAD67 (GABAergic interneurons); PV (parvalbumin-positive interneurons); SST (somatostatin-positive interneurons). AAV, adeno-associated virus; CBA, chicken β-actin promoter; chr2, chromosome 2; EGFP, enhanced green fluorescent protein; eTF, engineered transcription factor; hGH-pA, human growth hormone-derived poly-adenylation signal; IHC, immunohistochemistry; ICV, intracerebroventricular; ITR, inverted terminal repeat; ns, not significant; PND, postnatal day; RE^GABA^, GABAergic cell-selective regulatory element; SD, standard deviation; UTR, untranslated region; vg, vector genomes.

To selectively express the AAV9-RE^GABA^-eTF^*SCN1A*^ transgene in GABAergic inhibitory neurons, we developed a novel regulatory element cassette (RE^GABA^), designed from human-derived sequence features to collectively confer specificity to GABAergic interneurons. RE^GABA^ comprised enhancer, promoter, 5′UTR (untranslated region), and intronic sequences derived from the GABA-selective *hGAD1* gene locus, and a 3′UTR sequence designed to further reduce residual expression in excitatory neurons (Fig. 2A).^36,39,40^ These elements were designed to capture regulatory sequence features that confer endogenous cell selectivity in compact form, enabling cell-selective expression compatible with the 4.7 kb AAV packaging capacity.

Sequence selection was informed by genomic sequence conservation (Fig. 2B), and genomic regulatory element markers such as DNase I hypersensitivity peaks, epigenetic histone features, and transcription factor ChIP-seq clusters (Fig. 2B). The RE^GABA^ enhancer element is derived from a novel enhancer element located 50 kb upstream of the *GAD1* gene (Fig. 2A, B). The 3′UTR element comprised cognate target motifs for excitatory neuron-enriched miRNAs: miR128 and mir221,^36,39^ upstream of a human growth hormone poly-adenylation signal (Fig. 2A).

To evaluate the cell-selectivity of RE^GABA^ promoter *in vivo*, PND1 mice (*n* = 4/group) were dosed via bilateral ICV injection with 2.0E10 vg per animal of AAV9 with a chicken β-actin (CBA) promoter driving expression of EGFP [AAV9-CBA-EGFP], or EGFP expression driven by RE^GABA^ [AAV9-RE^GABA^-EGFP].

Quantitation of GFP colocalization with a subset of neuron-specific markers, including NeuN (neurons), CAMK2a (glutamatergic neurons), GAD67 (GABAergic interneurons), PV (parvalbumin-positive interneurons), and SST (somatostatin-positive interneurons), was then evaluated by IHC 27 days postinjection. AAV9-CBA-EGFP and AAV9-RE^GABA^-EGFP exhibited similar selectivity for expression in neurons (NeuN, ∼90% of expressing cells in both cases). However, AAV9-CBA-EGFP expression exhibited significantly higher selectivity for excitatory neurons and AAV9-RE^GABA^-EGFP showed higher selectivity of expression in GABAergic interneurons, as indicated by colocalization of EGFP and CAMK2a or GAD67 for excitatory neurons and GABAergic interneurons, respectively. Consistent with selectivity of AAV9-RE^GABA^-EGFP for GABAergic interneurons, RE^GABA^-driven EGFP had significantly higher colocalization with the GABAergic subtype markers PV and SST, compared with AAV9-CBA-EGFP (Fig. 2C).

We next evaluated the percentage of neuron marker-positive cells coexpressing EGFP. At a dose level of 2.0E10 vg per animal, AAV9-RE^GABA^-EGFP drove EGFP expression in 58.9% ± 14.4% GAD67-positive cells, 67.2% ± 18.7% PV-positive cells, and 82.0% ± 14.3% SST-positive cells, whereas AAV9-CBA-EGFP exhibited significantly lower expression coverage (Fig. 2D). Expression of GFP under the control of RE^GABA^ was limited to cells that coexpressed the GABAergic neuronal markers—GAD67, PV, and SST—while expression of GFP under the control of the constitutive CBA promoter was detected throughout the brain in multiple cell types (Fig. 2E). Minimal staining was observed in microglia and astrocytes (data not shown).

Taken together, these data demonstrate that the RE^GABA^ promoter element can significantly enhance selectivity of AAV9-driven transgene expression in GABAergic inhibitory interneurons via both a reduction in off-target expression within excitatory neurons and enhanced expression within GABAergic interneurons, compared with CBA.

### AAV9-RE^GABA^-eTF^*SCN1A*^ upregulates *SCN1A* in a cell-type selective manner *in vivo* with a corresponding increase in membrane-associated Na_V_1.1 protein

**Figure 3.**
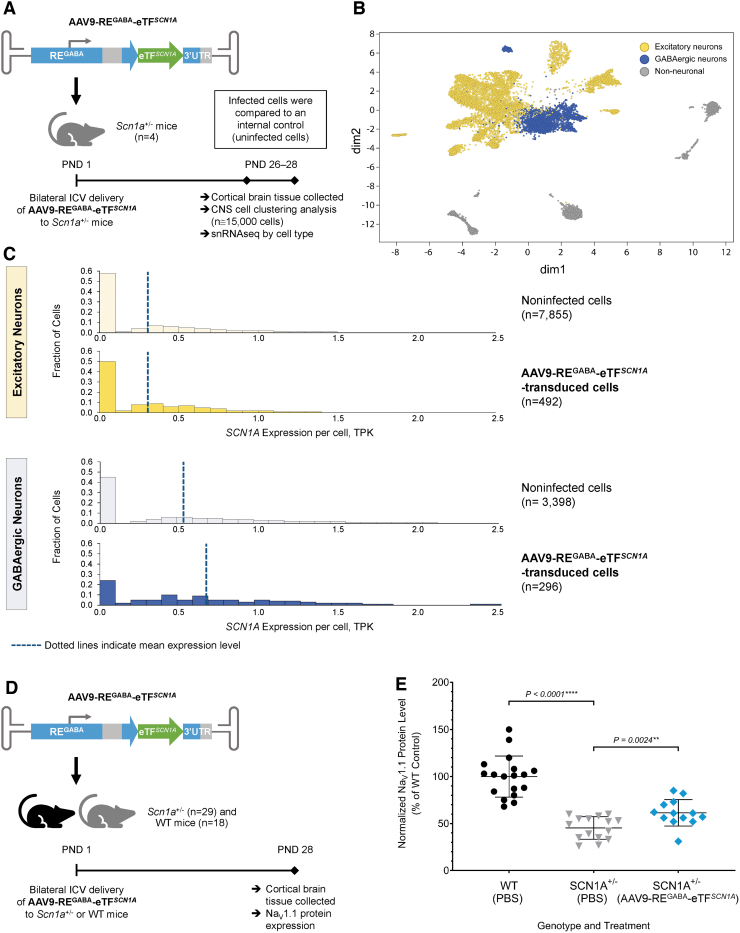
AAV9-RE^GABA^-eTF^*SCN1A*^ upregulates SCN1A within GABAergic inhibitory interneurons *in vivo*. **(A)** Experimental design to evaluate SCN1A expression in excitatory and GABAergic inhibitory neurons. Four *Scn1a*^+/−^ mice were treated with 1.7E10 vg per animal of AAV9-RE^GABA^-eTF^*SCN1A*^ on PND1 via bilateral ICV injection. On PND28, cortical brain tissue was collected, and clustering analysis was performed to classify over 15,000 cells into excitatory and inhibitory cell types based on transcriptome profiles. **(B)** Clustering of CNS cell types in cortical brain tissue. Gene expression visualization using tSNE to compute a low-dimensional representation of snRNAseq data; dim2 and dim1 are the outputs of tSNE, the 2D representation of the gene expression profiles of each cell. *Yellow* dots denote excitatory neurons; *blue* dots denote GABAergic inhibitory neurons; *gray* dots denote non-neuronal cells. **(C)** *SCN1A* mRNA transcript levels in excitatory and GABAergic inhibitory neurons. AAV9-RE^GABA^-eTF^*SCN1A*^ upregulates *SCN1A* mRNA specifically in GABAergic inhibitory neurons, with no upregulation in excitatory neurons. *Dotted lines* indicate mean SCN1A TPK per cell. **(D)** Experimental design to determine Na_V_1.1 protein expression. One-day-old (PND1) *Scn1a*^+/−^ mice were administered PBS (*n* = 16) or 5.1E10 vg per animal AAV9-RE^GABA^-eTF^*SCN1A*^ (*n* = 13) and 18 WT littermates were administered PBS via a bilateral ICV injection. Animals were sacrificed on PND28 and brain tissue was evaluated by MSD electrochemiluminescence-based sandwich immunoassay. **(E)** Na_V_1.1 protein expression. Membrane-associated Na_V_1.1 protein levels from PBS-treated WT (6 males/12 females; *black circle*) or *Scn1a*^+/−^ mice (7 males/9 females; *gray triangle*) and AAV9-RE^GABA^-eTF^*SCN1A*^-treated *Scn1a*^+/−^ mice (9 males/4 females; *blue diamond*). Mean levels ± SD are indicated; ****p < 0.0001 and **p = 0.0024, Mann–Whitney test. CNS, central nervous system; dim, dimension; MSD, Meso Scale Discovery; Na_V_1.1, voltage-gated type I sodium channel; PBS, phosphate-buffered saline; snRNAseq, single-nucleus RNA sequencing analysis; TPK, transcripts per 1000 total transcripts; tSNE, t-distributed stochastic neighbor embedding; WT, wild-type.

To determine *SCN1A* upregulation within GABAergic inhibitory interneurons, *Scn1a*^+/−^ mice (*n* = 4) received AAV9-RE^GABA^-eTF^*SCN1A*^ (1.7E10 vg/animal) via bilateral ICV injection on PND1 (Fig. 3A). On PND28, cortical brain tissue was collected and snRNAseq was performed from isolated cortical nuclei. Clustering analysis was performed to classify over 15,000 cells into excitatory/inhibitory neurons and non-neuronal cell types based on transcriptome profiles (Fig. 3B). Cell types were annotated based on canonical markers, as shown in the heat map (Supplementary Fig. S1).

*SCN1A* expression was detected in both excitatory and inhibitory cells (Fig. 3C, noninfected cells, pale yellow and pale blue histograms), similar to what has been reported by other groups.^41^ Overall, *SCN1A* transcript levels in cortical brain were ∼2-fold higher in GABAergic cells (dark blue histogram; average transcripts per 1000 total transcripts [TPK] 0.55) than in excitatory cells (dark yellow histogram; average TPK 0.31). GABAergic neurons containing eTF^*SCN1A*^ transcript expressed ∼30% more *SCN1A* than GABAergic cells that lacked eTF^*SCN1A*^ (Fig. 3C; blue panel, average TPK 0.69 vs. 0.54; *p* < 0.001; one-sided rank-sum test). Importantly, treatment with AAV9-RE^GABA^-eTF^*SCN1A*^ did not elevate *SCN1A* expression in excitatory cells (average TPK 0.31) over basal levels (yellow panel, *p* > 0.01) (Fig. 3C). These results indicate that AAV9-RE^GABA^-eTF^*SCN1A*^ results in selective upregulation of *SCN1A* in GABAergic inhibitory interneurons.

Upregulation of *SCN1A* led to a corresponding increase in Na_V_1.1 protein levels in brain tissue. In a separate experiment, *Scn1a*^+/−^ mice (*n* = 13) were administered AAV9-RE^GABA^-eTF^*SCN1A*^ (5.1E10 vg/animal) and as controls, *Scn1a*^+/−^ mice (*n* = 16) and WT littermates (*n* = 18) received PBS vehicle via ICV injection, and membrane Na_V_1.1 protein expression was measured by MSD electrochemiluminescence-based sandwich immunoassay (Fig. 3D). As expected, Na_V_1.1 protein expression in vehicle-treated *Scn1a^+/^*^−^ mice was significantly lower when compared with WT mice (45.7% ± 11.7% vs. 99.9% ± 21.8%, respectively; *p* < 0.0001). AAV9-RE^GABA^-eTF^*SCN1A*^ resulted in the upregulation of membrane-associated Na_V_1.1 protein in the CNS tissue of *Scn1a^+/^*^−^ mice by ∼30% compared with vehicle-treated *Scn1a^+/^*^−^ animals (61.4% ± 14.1% vs. 45.7% ± 11.7%, respectively; *p* = 0.0024) (Fig. 3E).

### AAV9-RE^GABA^-eTF^*SCN1A*^ reduces the frequency, duration, and severity of spontaneous seizures and rescues HTS sensitivity in Dravet mice

We next tested the potential for AAV9-RE^GABA^-eTF^*SCN1A*^ treatment to reduce seizures in the DS mouse model. The optimal interventional timepoint for dosing was determined based on the kinetics of AAV9 transgene expression which requires ∼30 days to reach stable expression levels (Supplementary Fig. S2). Considering that the onset of seizures and mortality in *Scn1a*^+/−^ mice occurs between the third and fourth weeks of life,^17,24,42^ PND1 was determined to be the optimal time for dosing as the stabilization of transgene expression at approximately PND30 occurs following onset of seizure symptoms but before high rates of animal attrition due to SUDEP.

**Figure 4.**
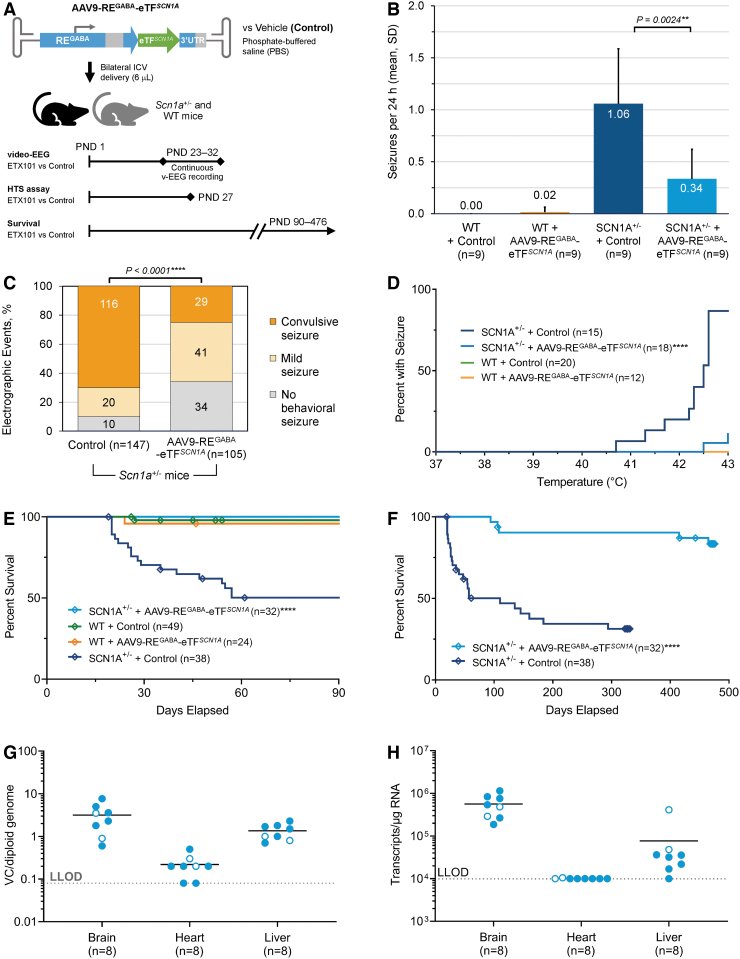
AAV9-RE^GABA^-eTF^*SCN1A*^ reduces frequency and severity of spontaneous seizures, protects against febrile seizures, and demonstrates durable survival efficacy and persistent activity for up to 470 days postdosing in *Scn1a*^+/−^ mice. **(A)** Study design. AAV9-RE^GABA^-eTF^*SCN1A*^ or vehicle alone (PBS) were administered by bilateral ICV injection (3 μL/hemisphere) to heterozygous *Scn1a*^+/−^ or WT littermates at PND1. Separate studies assessed electrographic seizure monitoring of spontaneous seizures (following doses of 1.2–5.4E11 vg/animal^a^ vs. vehicle); susceptibility to HTS (3.7E10 vg/animal^a^ vs. vehicle); and survival and long-term persistence of AAV9-RE^GABA^-eTF^*SCN1A*^ (3.7E10 vg/animal^a^ vs. vehicle). At 470 days, the animals were sacrificed, and brain, heart, and liver tissues were evaluated for AAV9-RE^GABA^-eTF^*SCN1A*^ VCN by ddPCR and eTF transgene mRNA transcript levels by RT-ddPCR. **(B)** EEG seizure frequency. Mice treated with AAV9-RE^GABA^-eTF^*SCN1A*^ experienced a 68% reduction in the mean daily generalized seizure frequency per animal compared with *Scn1a*^+/−^ controls (**p = 0.0024, unpaired *t-*test). No changes in seizure frequency were detected in WT mice treated with AAV9-RE^GABA^-eTF^*SCN1A*^. **(C)** EEG seizure severity. Video characterization of all recorded electrographic seizure events revealed that treatment of *Scn1a*^+/−^ mice with AAV9-RE^GABA^-eTF^*SCN1A*^ significantly reduced convulsive (tonic-clonic) seizures compared with *Scn1a*^+/−^ mice treated with vehicle (****p < 0.0001; chi-square test). Most events detected in AAV9-RE^GABA^-eTF^*SCN1A*^-treated *Scn1a*^+/–^ mice were mild (characterized by mild movements and/or head twitching, but without convulsions) or nonbehavioral seizures. **(D)** HTS assay. Percentage of AAV9-RE^GABA^-eTF^*SCN1A*^ PND1-treated *Scn1a*^+/−^ mice experiencing seizures at a given temperature at PND27. WT groups overlap along the zero line (*green* and *orange* lines; only the *orange* trace is shown). *p*-Values calculated using a Log-rank test, ****p < 0.0001. **(E)** Ninety-day survival. The 90-day survival rate in AAV9-RE^GABA^-eTF^*SCN1A*^-treated *Scn1a*^+/–^ mice was 100% compared with 50% in control-treated *Scn1a*^+/−^ mice (****p < 0.0001, Log-rank test). No difference in survival was observed between WT mice ± AAV9-RE^GABA^-eTF^*SCN1A*^ and *Scn1a*^+/−^ mice + AAV9-RE^GABA^-eTF^*SCN1A*^. **(F)** Long-term Survival. Long-term follow-up showed that survival benefit of AAV9-RE^GABA^-eTF^*SCN1A*^ was sustained over ∼470 days after dosing (****p < 0.0001, Log-rank test). *Diamond* indicates humane endpoint or end of study euthanasia at approximately day 330 for vehicle and day 470 for AAV9-RE^GABA^-eTF^*SCN1A*^-treated animals. **(G)** VCN biodistribution in *Scn1a*^+/−^ mice administered AAV9-RE^GABA^-eTF^*SCN1A*^. At 470 days, the animals were sacrificed, and brain, heart, and liver tissues were evaluated for AAV9-RE^GABA^-eTF^*SCN1A*^ VCN by ddPCR and eTF^*SCN1A*^ transgene mRNA transcripts levels by RT-ddPCR. **(H)** eTF^*SCN1A*^ mRNA expression levels in *Scn1a*^+/−^ mice administered AAV9-RE^GABA^-eTF^*SCN1A*^. eTF^*SCN1A*^ mRNA transcripts per microgram of RNA analyzed. Seven of eight animals had no measurable eTF^*SCN1A*^ mRNA transcripts in the heart. *Open circle* indicates female gender. *Dotted line* indicates the limit of detection in both assays. ^a^Doses in the EEG study were determined by a qPCR titering method while the HTS and survival studies were titered via ddPCR; thus the doses used across studies are not directly comparable. EEG, electroencephalography; HTS, hyperthermia-induced seizure; LLOD, lower limit of detection; qPCR, quantitative polymerase chain reaction; RT-ddPCR, reverse transcription digital droplet polymerase chain reaction; VCN, vector copy number.

To evaluate spontaneous seizures, we dosed WT (*n* = 10 vehicle, *n* = 7 AAV9-RE^GABA^-eTF^*SCN1A*^) and *Scn1a*^+/−^ (*n* = 17 vehicle, *n* = 18 AAV9-RE^GABA^-eTF^*SCN1A*^) mice at PND1 via bilateral ICV injection (1.2–5.4E11 vg/animal; 3 μL/hemisphere). Animals were monitored continuously by video-EEG for occurrence of electrographic seizures from PND21 for 9 days. For HTS, we dosed WT (*n* = 20 vehicle, *n* = 12 AAV9-RE^GABA^-eTF^*SCN1A*^) and *Scn1a*^+/−^ (*n* = 15 vehicle, *n* = 18 AAV9-RE^GABA^-eTF^*SCN1A*^) mice at PND1 with a total 3.7E10 vg per animal. HTS assays were performed on PND 27 (±1 day) (Fig. 4A).

Treatment of *Scn1a*^+/−^ mice with AAV9-RE^GABA^-eTF^*SCN1A*^ at PND1 significantly reduced the frequency and severity of spontaneous seizures and increased the number of seizure-free mice over a 9-day assessment period. AAV9-RE^GABA^-eTF^*SCN1A*^ treatment reduced average number of daily seizures by 68% (*p* = 0.0024, unpaired *t*-test), whereas no differences in seizure frequency were detected in WT mice treated with vehicle or AAV9-RE^GABA^-eTF^*SCN1A*^ (Fig. 4B). In addition, blinded video-EEG assessment revealed that electrographic seizure events experienced by *Scn1a*^+/−^ mice treated with AAV9-RE^GABA^-eTF^*SCN1A*^ were less likely to manifest as convulsive seizure events, compared with *Scn1a*^+/−^ mice treated with vehicle (*p* < 0.0001, chi-square test, Fig. 4C). Finally, the percentage of seizure-free mice increased from 20% in vehicle-treated mice to 67% in AAV9-RE^GABA^-eTF^SCN1A^-treated mice.

In the HTS assay (Fig. 4D), vehicle-treated *Scn1a*^+/−^ mice exhibited temperature-induced seizures, with ∼87% exhibiting seizures at an internal body temperature of 43.0°C. Treatment of *Scn1a*^+/−^ mice at PND1 with 3.7E10 vg per animal AAV9-RE^GABA^-eTF^*SCN1A*^ significantly increased the temperature threshold for developing HTS (*p* < 0.0001, Log-rank test). Approximately 88% of AAV9-RE^GABA^-eTF^*SCN1A*^-treated *Scn1a*^+/−^ mice remained seizure free at 43.0°C. No seizures were observed in WT mice treated with vehicle or AAV9-RE^GABA^-eTF^*SCN1A*^. Similarly, treatment with AAV9-RE^GABA^-eTF^*SCN1A*^ also significantly increased resistance to HTS in an alternative mouse model of DS,^11,25^ where a nonsense mutation (R1407X) was introduced in the reading frame of one copy of *SCN1A* (Supplementary Fig. S3A).

### AAV9-RE^GABA^-eTF^*SCN1A*^ extends survival in Dravet mice up to 470 days postdosing and persists preferentially in the brain

The ability of AAV9-RE^GABA^-eTF^*SCN1A*^ to prolong short-term and long-term survival was assessed in *Scn1a*^+/−^ mice injected at PND1 with vehicle or AAV9-RE^GABA^-eTF^*SCN1A*^ (3.7E10 vg/animal; *n* = 70) and followed for 90 days or 470 days. As controls, WT littermates were administered PBS or AAV9-RE^GABA^-eTF^*SCN1A*^ (3.7E10 vg/animal; *n* = 73 at PND1) (Fig. 4A).

After 90 days, 100% of *Scn1a*^+/−^ mice treated with AAV9-RE^GABA^-eTF^*SCN1A*^ were alive compared with 50% of vehicle-treated *Scn1a*^+/−^ mice (Fig. 4E; *p* < 0.0001, Log-rank test). There were no differences in survival between WT mice treated with AAV9-RE^GABA^-eTF^*SCN1A*^ or vehicle. At 470 days, AAV9-RE^GABA^-eTF^*SCN1A*^-treated *Scn1a*^+/−^ mice showed extended survival (*n* = 32; 83.2%) when compared with vehicle-treated *Scn1a*^+/−^ mice (*n* = 38; 31.4%; Fig. 4F; *p* < 0.0001, Log-rank test). Similarly, treatment with AAV9-RE^GABA^-eTF^*SCN1A*^ also significantly prolonged survival in the alternative *Scn1a*^+/R1407X^ mouse model of DS (Supplementary Fig. S3B).

AAV9-RE^GABA^-eTF^*SCN1A*^ VCN and eTF^*SCN1A*^ transcript levels were measured in brain, heart, and liver of *Scn1a*^+/−^ mice (*n* = 8) at day 470 postdosing. We found vector persistence in the brain, with a mean of 3.2 vector copies/diploid genome (Fig. 4G), while lower levels were detected in liver and heart (mean 1.3 and 0.2 vector copies/diploid genome, respectively). Similarly, eTF^*SCN1A*^ transcript levels were measured in the brain at a mean of 5.6E5 transcripts per microgram of RNA (Fig. 4H), indicating persistent mRNA expression. eTF^*SCN1A*^ mRNA in the liver was 7.5-fold lower (mean 7.5E4 transcripts/μg RNA); and seven of eight animals had no measurable eTF^*SCN1A*^ transcripts in the heart. One animal with detectable mRNA in the heart had a level of 1.05E4 transcripts per microgram RNA (Fig. 4H).

Collectively, these results show AAV9-RE^GABA^-eTF^*SCN1A*^ persistently and preferentially targets brain tissue in *Scn1a*^+/−^ mice and is associated with extended survival up to 470 days.

### Unilateral ICV administration of AAV9-RE^GABA^-eTF^*SCN1A*^ in NHPs resulted in widespread and selective CNS biodistribution and was well tolerated over 28 days

**Figure 5.**
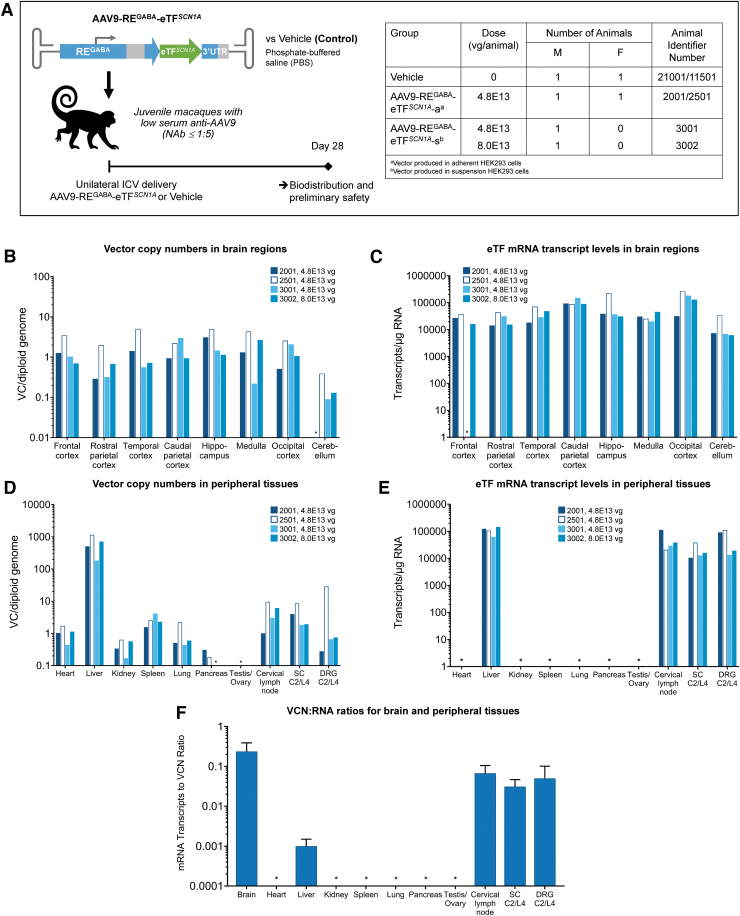
Unilateral ICV delivery of AAV9-RE^GABA^-eTF^*SCN1A*^ to NHPs leads to widespread vector biodistribution and increased eTF^*SCN1A*^ mRNA transcript levels in the brain with low off-target vector expression in peripheral tissues of NHPs. **(A)** Study design. AAV9-RE^GABA^-eTF^*SCN1A*^ (4.8–8.0 E13 vg/animal) or vehicle alone (PBS) was administered by unilateral ICV injection to four juvenile cynomolgus macaques (3M and 1F). All animals were sacrificed 28 ± 2 days after injection. Biodistribution of AAV9-RE^GABA^-eTF^*SCN1A*^ vector copies and expression of transgene mRNA were measured in target neuronal and peripheral tissues using ddPCR **(B–E)**. **(B)** AAV9-RE^GABA^-eTF^*SCN1A*^ vector biodistribution in brain regions. **(C)** eTF^*SCN1A*^ mRNA expression levels in brain regions. **(D)** AAV9-RE^GABA^-eTF^*SCN1A*^ vector biodistribution and **(E)** eTF^*SCN1A*^ mRNA expression levels in peripheral tissues. **(F)** VCN:RNA ratios for brain and peripheral tissues (*N* = 4, mean values; error bars indicate SD). Starred organs (*) indicate VCN or RNA levels below the limit of detection of the assay. DRG, dorsal root ganglia; NAb, neutralizing antibodies; NHP, nonhuman primate; SC, spinal cord.

Safety, biodistribution, and activity of AAV9-RE^GABA^-eTF^*SCN1A*^ using unilateral ICV injection were evaluated in a pilot, non-GLP, 28-day study in juvenile NHPs (24–27 months of age) (Fig. 5A). Previously, we have demonstrated that unilateral ICV administration of AAV9 in NHPs results in widespread and symmetrical distribution throughout the brain.^43^ Animals received infusions of vehicle (*n* = 2), or AAV9-RE^GABA^-eTF^*SCN1A*^ at 4.8E13 vg per animal (*n* = 3) and 8.0E13 vg per animal (*n* = 1); no steroids or other immunosuppressant agents were administered. Because the difference between the two doses (4.8E13 and 8.0E13) of AAV9-RE^GABA^-eTF^*SCN1A*^ tested was less than twofold and led to no systematic differences in VCN or RNA transcript levels in the brain, the VCN and RNA expression data were combined for analysis (*n* = 4 animals).

ddPCR analysis of target neuronal and peripheral tissues showed that one-time administration of AAV9-RE^GABA^-eTF^*SCN1A*^ led to widespread vector biodistribution and robust transgene expression throughout the brain (Fig. 5B, C), including key structures involved in epilepsy and cognitive deficits in DS, such as cerebral cortex and hippocampus.^44^ The mean VCN and eTF^*SCN1A*^ mRNA transcripts in the forebrain regions, including frontal cortex, temporal cortex, parietal cortex, occipital cortex and hippocampus, were measured at 1.73 copies per diploid genome and 1.71E4 transcripts per microgram RNA, respectively.

VCN and RNA expression in the medulla were similar to that of forebrain regions, with mean values of 2.14 copies/diploid genome and 3.04E4 transcripts per microgram RNA, respectively. Vector distribution was ∼10-fold lower in the cerebellum than other brain regions tested (0.16 copies/diploid genome). However, RNA expression levels were similar (1.38E4 transcripts/microgram RNA), likely due to the abundance of GABAergic Purkinje cells and inhibitory interneurons present in cerebellum.^45^

Vector distribution was detected in most peripheral tissues examined (Fig. 5D, E), the liver showing the highest levels. However, eTF^*SCN1A*^ transgene expression in non-neuronal tissues was detected only in liver and cervical lymph nodes. Relative to VCN, average eTF mRNA levels were lower in the liver, DRG, spinal cord, and cervical lymph nodes by ∼200-, 5-, 8-, and 3.6-fold, respectively, compared with CNS tissues (Fig. 5F), indicating the selectivity of RE^GABA^ for CNS tissues.

AAV9-RE^GABA^-eTF^*SCN1A*^ was well tolerated with no adverse events occurring during administration and no detectable changes in clinical observations, body weight, or body temperature during the 28-day study. All animals survived until necropsy at 28 days. Histopathological evaluation revealed no adverse macroscopic or microscopic findings in the tissues examined, including brain, DRG, spinal cord, and non-neuronal tissues (heart, lungs, spleen, liver, and gonads). No DRG-related toxicity was observed. Blood chemistry was unchanged, except for minimum to mild transient elevations in alanine aminotransferase and aspartate aminotransferase on day 8 in AAV9-RE^GABA^-eTF^*SCN1A*^-treated animals, which was fully resolved without treatment by day 15 or day 22. No corresponding microscopic finding was noted in the liver for any animal.

All animals had a measurable amount of serum anti-AAV9 capsid neutralizing antibodies 2 weeks after vector administration, determined by methods described in the Supplementary Materials. Titers ranged from 1:135 to 1:405 and were sustained through the end of the study (Supplementary Table S4). One animal had a positive anti-eTF^*SCN1A*^-binding antibody titer (1:400) in serum at 4 weeks postdosing (Supplementary Table S5). None of the animals had quantifiable levels of anti-AAV9 or anti-eTF^*SCN1A*^ antibody response in the cerebrospinal fluid (CSF). No correlation was observed between the presence of serum antibody and AAV9-RE^GABA^-eTF^*SCN1A*^ VCN or eTF^*SCN1A*^ transcript levels in the brain or peripheral organs.

## DISCUSSION

Key limitations of conventional AAV-mediated gene replacement therapy have precluded DS from treatment by this modality, notably (1) transgene size restrictions due to the relatively small packaging capacity of AAV particles and (2) lack of cell type specificity to restrict transgene expression to target neurons following local CNS delivery.^46,47^

**Figure 6.**
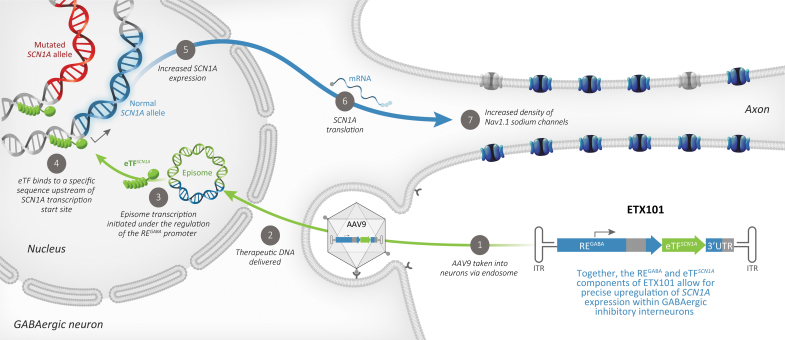
Mechanism of action of AAV9-RE^GABA^-eTF^*SCN1A*^ (ETX101), a cell-selective AAV-mediated *SCN1A* gene regulation therapy that upregulates expression of WT *SCN1A* selectively in GABAergic inhibitory interneurons to compensate for the loss-of-function mutant alleles in individuals with DS. As previously described,^46^ AAV vector enters the cell by endocytosis in a receptor-mediated manner. Vector escapes from the endosome, enters the nucleus through the nuclear pore complex, and predominantly exists as a nonreplicating episome. Episome transcription is then initiated under the regulation of the RE^GABA^ promoter to produce eTF^*SCN1A*^ preferably in GABAergic neurons. eTF^*SCN1A*^ binds to a conserved regulatory region upstream of the *SCN1A* transcription start site, promoting increased *SCN1A* expression and protein translation, thereby increasing the density of membrane-associated Na_V_1.1 sodium channels and restoring function. While eTF^*SCN1A*^ also binds the mutated *SCN1A* allele, it does not produce any stable protein capable of functioning at the neuronal membrane. This approach leverages natural patterns of gene expression to increase production of Na_V_1.1 protein and restore inhibitory function while minimizing potential off target effects. DS, Dravet syndrome; RE, regulatory element.

To overcome these key limitations, we developed AAV9-RE^GABA^-eTF^S*CN1A*^, a novel gene regulation therapy, to selectively drive upregulation of endogenous WT *SCN1A* in GABAergic inhibitory interneurons to rescue haploinsufficiency of *SCN1A* in DS (Fig. 6). Using a compact engineered transcription factor (eTF^*SCN1A*^) to selectively target a unique and conserved genomic *SCN1A* regulatory sequence, we generate potent and specific upregulation in *SCN1A* mRNA expression *in vitro* and *in vivo.* Dynamic changes in Na_V_1.1 expression and localization can be influenced by intrinsic regulatory mechanisms—alternative splicing, trafficking, and post-translational modifications—that provide control mechanisms to regulate the appropriate balance of neuronal excitability.^48,49^

Our strategy regulates endogenous *SCN1A* transcription upstream of these intrinsic regulatory mechanisms, which may provide additional physiological control, and safeguards against overexpression. Our results show that a one-time ICV administration of AAV9-RE^GABA^-eTF^SCN1A^ is well tolerated in NHPs and significantly prolongs survival, and significantly reduces both spontaneous seizures and HTS in a mouse model of DS.

In the brain, Na_V_1.1 is predominantly expressed in GABAergic inhibitory interneurons, where it maintains the appropriate balance of inhibitory neurotransmission. Multiple lines of evidence have established impaired excitability of GABAergic inhibitory interneurons due to Na_V_1.1 haploinsufficiency as fundamental in driving key DS phenotypes, and suggest that enhancement of GABA signaling may improve seizure control and other manifestations associated with DS.^12,13,15–21^ On the contrary, pan-neuronal overexpression of Nav1.1 in both GABAergic inhibitory interneurons and excitatory neurons would lead to an opposing physiological effect, increasing the excitability of both cell types and further disrupting the balance of excitation and inhibition.^50–53^

Accordingly, we designed an expression cassette (collectively referred to as RE^GABA^) to drive potent expression in multiple subtypes of GABAergic interneurons, with minimal expression in excitatory neurons, by utilizing optimized human genomic regulatory sequences surrounding the *GAD1* gene locus, combined with a 3′ excitatory detargeting UTR sequences.^39^

When compared with the constitutive CBA promoter, we show that expression of eTF^*SCN1A*^ under the control of RE^GABA^ upregulates *SCN1A* expression specifically within GABAergic neurons *in vivo*, resulting in ∼30% elevation in Na_V_1.1 protein levels. This level of increase was sufficient to rescue multiple disease phenotypes in a DS mouse model, highlighting the importance of cell-type selectivity. In addition, we detected low levels of transgene expression in peripheral tissues of NHPs, including the liver. This is in contrast to the finding that intrathecal delivery of AAV vectors to the CSF still results in peripheral transgene expression.^54^ By emulating endogenous expression patterns and gene regulation mechanisms, we can avoid supraphysiological protein expression and improve safety and potency of gene therapies. Beyond DS, GABAergic dysfunction has been implicated in other autism spectrum disorder-related syndromes, including Angelman syndrome, Rett syndrome, and others.^55–59^

Thus, targeted gene delivery to GABAergic inhibitory interneurons may have the potential to address the neurocircuitry deficits observed across multiple neurodevelopmental and epileptic encephalopathies. Our results provide proof-of-concept for future targeted gene delivery strategies for other interneuronopathies.

DS has been successfully recapitulated in well-characterized mouse models using several genetic strategies, including heterozygous *Scn1a*^+/−^ models, the R1407X knock-in model resulting in a truncated α-subunit, and others.^11,12,24^ Importantly, the *Scn1a*^+/−^ model recapitulates seizure-modifying efficacy for several treatments clinically indicated for DS patients. Hawkins *et al.*^60^ used the *Scn1a*^+/−^ model to evaluate the efficacy of multiple antiseizure medications approved for DS, including clobazam, topiramate, stiripentol, and valproic acid. In this study, the authors found no correlation between seizure reduction (as measured by the HTS assay) and extended survival, even at supratherapeutic doses of these compounds.^60^

In contrast, we demonstrate that a single bilateral ICV administration of AAV9-RE^GABA^-eTF^S*CN1A*^ at PND1 leads to both a significant reduction in HTS sensitivity and extended survival in two distinct genetic mouse models of DS (*Scn1a*^+/−^ and *Scn1a*^+/R1407X^). We also show that severity of spontaneous seizures as measured by EEG activity is significantly reduced in *Scn1a*^+/−^ mice.

Importantly, reduction of sensitivity to HTS was also observed when mice were dosed as late as PND5, indicating a potentially broad effective treatment window of AAV9-RE^GABA^-eTF^S*CN1A*^ (Supplementary Fig. S4). In support of this, a recent study showed that reactivation of *Scn1a* expression in a *Scn1a* conditional knock-in mouse model can rescue seizure activity and behavioral abnormalities, and normalize interneuron excitability, even months after symptom onset.^61^ Together, these data suggest that disease phenotype may be reversed and strongly support continued development of AAV9-RE^GABA^-eTF^S*CN1A*^ as a novel gene therapy with the potential to durably rescue multiple phenotypes in DS patients.

In AAV-mediated gene therapy, the careful selection of the proper route of administration is important as it can influence biodistribution, efficacy, and safety. Intravenous administration for systemic exposure to multiple organs usually requires larger doses, while local administration to smaller organs, such as the eye and brain, requires smaller doses for transduction of relevant cells and may limit systemic exposure. CNS-administered investigational AAV-mediated gene therapies are in development for multiple CNS disorders and use different local routes, including intrathecal, intraparenchymal, intracisterna magna, and ICV. Because these routes show different AAV biodistribution profiles in the brain, the decision on the optimal one is intimately related to the underlying biology of the specific disease and affected areas.^62^

Previously, we demonstrated that unilateral ICV administration of AAV9 in NHPs results in widespread and symmetrical distribution throughout the brain, with significantly improved transduction of forebrain structures compared to intrathecal-lumbar administration.^43^ ICV is a well-established and widely used method of drug delivery for the treatment of pediatric and adult patients with a broad range of diseases.^63^ Here, we show that a one-time, unilateral ICV injection of AAV9-RE^GABA^-eTF^*SCN1A*^ in NHPs was well tolerated and led to widespread vector biodistribution and robust transgene expression throughout the brain. These results confirm ICV as an efficient and safe delivery route for AAV9-RE^GABA^-eTF^*SCN1A*^, reaching areas of the brain associated with DS manifestations that are not currently addressed by antiseizure medications.

Several potential disease-modifying therapies for DS are under various stages of development,^64–69^ which differ in their approaches and neuronal cell types targeted. For example, a non-GABA selective, antisense oligonucleotide-based therapy has been reported to be well tolerated in DS patients at multiple monthly doses of up to 20 mg.^66,69^ The safety, pharmacokinetic profile, and efficacy of higher doses under placebo-controlled conditions will determine the frequency of intrathecal injections required to maintain therapeutic effect.^70,71^

Importantly, AAV9-mediated gene therapies have shown transgene persistence after a single administration.^72^ AAV-delivered transgenes form stable, nonreplicating circular episomal DNA, which is gradually lost in dividing cells, but persists in postmitotic cells, such as neurons, due to the very low cellular turnover.^46^ Recently published AAV-mediated gene therapies demonstrate compelling proof of concept, but are limited in their potential for clinical development. A GABA-targeted gene therapy strategy for DS utilizing a Na_V_1.1 channel modulator (AAV-Navβ1) reported only partial phenotypic rescue, with no effect on febrile seizure susceptibility, and limited GABA specificity.^65^

Conditional Cas9-based transcriptional activators, combined with GABA-specific Cre mouse lines or codelivery of a targeting vector emphasize the potential for a GABA-targeted *SCN1A* gene regulation in treating cognitive and behavioral effects, improving survival rate, and reducing seizure susceptibility.^64,68^ However, GABA-selective expression of these described transgenes is required for therapeutic efficacy, and the size of these effector transgenes precludes packaging within a single AAV particle.^49,53^ AAV9-RE^GABA^-eTF^S*CN1A*^ achieves GABA-specific expression of an *SCN1A* transcriptional activator using a single AAV vector, enabling a clinically tractable path for an AAV-mediated gene therapy for DS.

## CONCLUSIONS

Our preclinical data demonstrate the efficacy and preliminary safety of AAV9-RE^GABA^-eTF^*SCN1A*^ for selectively increasing *SCN1A* gene activity and Na_V_1.1 protein expression in the brain. This resulted in reduced frequency of spontaneous seizures and HTS and increased long-term survival in a robust and translatable mouse model of SCN1A^+^ DS. To our knowledge, this is the longest follow-up reported in the DS mouse model, with unprecedented survival achieved after a single treatment administration. The duration of AAV9-RE^GABA^-eTF^S*CN1A*^ expression in CNS in humans is not yet quantified, but early results on the durability of AAV-mediated gene therapy in other indications have been positive, with a persistence of treatment effect reported in patients with SMA for over 6 years.^73^ Preliminary safety findings in juvenile NHPs support the feasibility of AAV9-RE^GABA^-eTF^*SCN1A*^ delivery by one-time unilateral ICV administration.

Collectively, these preclinical findings establish strong proof-of-concept for an AAV9-mediated gene regulation therapy that targets GABAergic inhibitory neurons to address the underlying cellular and genetic mechanism of SCN1A^+^ DS. Current treatments for this devastating condition provide only partial control of select aspects of the DS phenotype, underscoring the need for disease-modifying therapies. This unmet need supports the ongoing clinical development of AAV9-RE^GABA^-eTF^*SCN1A*^ (ETX101) to address the full spectrum of SCN1A^+^ DS manifestations.

## Supplementary Material

Supplemental data
