# Supplementary material for: Cell-Selective Adeno-Associated Virus-Mediated SCN1A Gene Regulation Therapy Rescues Mortality and Seizure Phenotypes in a Dravet Syndrome Mouse Model and Is Well Tolerated in Nonhuman Primates
Source: Hum Gene Ther. 2022 Jun 10;33(11-12):579–97. doi: 10.1089/hum.2022.037 (PMC9242722; doi:10.1089/hum.2022.037)

SUPPLEMENTARY MATERIAL

Supplementary methods S2

Immunohistochemistry image analysis S2

Serum and CSF anti-AAV9 and anti-eTF antibody assays S2

Supplementary Table S1. Primers for RT-qPCR in vitro S3

Supplementary Table S2. Primers for genotyping S3

Supplementary Table S3. eTF^{SCN1A} binding site sequence homology: BLASTn results for eTF^{SCN1A} target sequence in human genome. S4

Supplementary Table S4. Pre- and post-dose anti-AAV9 neutralizing antibody (NAb) titer in cynomolgus monkey serum and CSF. S5

Supplementary Table S5. Anti-eTF^{SCN1A} antibody titers in cynomolgus monkey serum and CSF. S6

Supplementary Figure S1. Clustering analysis of CNS cell types in cortical brain tissue. S6

Supplementary Figure S2. Kinetics of vector expression in mouse brain following ICV delivery of AAV9-eGFP. S8

Supplementary Figure S3. Hyperthermia-induced seizures and long-term survival in *Scn1a*$^{+/R1407X}$ Dravet mice. S9

Supplementary Figure S4. Hyperthermia-induced seizures and 90-day survival in *Scn1a*$^{+/-}$ and WT mice dosed at PND5. .... S10

Supplementary methods

Immunohistochemistry image analysis

A 1-mm square region of the somatosensory cortex was selected from each animal. This raw data image was spectrally unmixed with Akoya InForm software using a spectral library constructed from single stained slides stained in parallel in the same assay. A separate spectral library was generated and used for the first and second multiplex data sets. A binary image of the nuclei in the DAPI channel was created manually from all images from the first data set (4 test and 4 control). These 'ground-truth' binary masks were used to train a StarDist neural network segmentation model which was used with the Fiji plugin to perform nuclei segmentation for all the DAPI-stained images.[1] Validation of this model using a 'ground-truth' image not part of the original training set yielded a precision and recall of 98% each. In Fiji, once nuclei regions were identified they were overlayed on each marker image and regions were visually identified as positive or negative for a respective marker. During region identification each marker image was inspected separately and not overlaid with any other marker. Inspection was performed on a 27" 4K UHD monitor with the image zoom increased to 200%. Images were grayscale and not contrast adjusted. Counts per image for nuclear region GFP or marker status were found and percentages were calculated using the total nuclei count. The ratio of percent double-positive GFP with a respective marker divided by percent total GFP positive was found for each marker in addition to the ratio of double-positive divided by percent total marker positive regions.

Serum and CSF anti-AAV9 and anti-eTF antibody assays

Anti-eTF^{SCN1A} binding IgG antibodies were determined by ELISA. Briefly, 96-well High Binding ELISA microplates were coated with 100 µL/well of recombinant eTF^{SCN1A} (a transgene encoded protein, diluted in 50 mM carbonate-bicarbonate buffer, pH 9.6) and incubated at 4°C overnight. Plates were washed with 1X PBS-T) buffer and blocked with 3% bovine serum albumin in PBS for 1 hour. One hundred microliters of diluted serum or CSF sample was added to each well and incubated for 1 hour. After wash, detection antibody (HRP-Goat anti-Monkey IgG) was added, incubated for 1 hour, and washed. The color was developed by adding 100 µL of TMB Substrate Solution to each well for 3 minutes and followed by 100 µL of stop solution. The optical density (OD) was measured at 450/640 nm.

For the anti-AAV9 neutralizing antibody assay, heat-inactivated sera or CSF samples were continuously diluted 2-fold with Dulbecco's Modified Eagle Medium supplemented with 10% heat-inactivated fetal bovine serum and preincubated with AAV9-CMV-NLuc vector at 37°C for 60 minutes. Twenty-five microliters of the mixture were added to 96-well plates containing 1.5E4 of 293AAV cells to reach a final multiplicity of infection of 10,000 vg/cell (titer determined by qPCR), followed by an incubation at 37°C in a 5% CO_2 incubator for 48 hours. Luciferase expression was measured with a luciferase assay kit (Promega, Madison, WI, USA). The neutralizing antibody titer was determined as the serum dilution at which 50% of AAV reporter gene expression is permitted, compared to no serum controls.

1. Schmidt U, Weigert M, Broaddus C, Myers G. Cell detection with star-convex polygons. In: Frangi AF, Schnabel JA, Davatzikos C, López Alberola C, Fichtinger G, editors. Medical Image Computing and Computer Assisted Intervention – MICCAI 2018 (21st International Conference, Granada, Spain, September 16-20, 2018, Proceedings, Part II): Springer International Publishing; 2018. p. 265-73.

Supplementary Table S1. Primers for RT-qPCR in vitro

**Target gene**	**Forward primer**	**Reverse primer**
SCN1A	5′-TGTCTCGGCATTGAGAACATTC-3′	5′-ATTGGTGGGAGGCCATTGTAT-3′
SCN9A	5′-AAGCCCCAAAGCCAAGCAG-3′	5′-AGGTGTGGCATTGAAACGG-3′
TTC21B	5′-GGTCACGTACAGCTTCGCAT-3′	5′-CTGGTTTCTGGCTCGTGGAG-3′
UTRN	5′-TGACAATGGGCAGAACGAAT-3′	5′-TGCAGCACTCTGTTGACGTT-3′
GRN	5′-ATGGTCAGTTCTGCCCTGTG-3′	5′-CGGTAAAGATGCAGGAGTGGC-3′
SNCA	5′-CCTTCTGCCTTTCCACCCT-3′	5′-TCCCTCCTTGGCCTTTGAAA-3′
GAPDH	5′-ACCACAGTCCATGCCATCAC-3′	5′-TCCACCACCCTGTTGCTGTA-3′

Supplementary Table S2. Primers for genotyping

**SCN1A Forward primer**	5′-GCACAGATATATGTGTGAAATTTGAGTTCT-3′
**SCN1A Reverse primer**	5′- CGAAACAATTCTGATACATGGGAGAGT-3′
**SCN1A reporter 1 sequence**	5′- ATGCATCTTGGATTTGGCC -3′
**WT Forward primer**	5′- GGCACAGATATATGTGTGAAATTTGAGTT -3′
**WT Reverse primer**	5′- CGATATCAAGCTTACTAGTATAACTTCGTATAGC -3′
**WT reporter 1 sequence**	5′- AAAGGATGCATATCGATTACC -3′

Supplementary Table S3. eTF^{SCN1A} binding site sequence homology: BLASTn results for eTF^{SCN1A} target sequence in human genome.

Nearest gene (<100kb)	Strand	Length of match (bp)	E-val	Annotation	Distance to TSS (bp)	Overlap with FANTOM5 annotations
SCN1A	-	18	0.098	promoter-TSS (NM_001202435)	1	SCN1A promoter
none	+	17	0.39	Intergenic	209794	No
TENM3	-	16	1.5	intron (NM_001080477, intron 3 of 27)	133813	No
PLSCR5	+	15	6.1	Intergenic	-41225	No
ADCY2	+	15	6.1	intron (NM_020546, intron 2 of 24)	38425	No
TINAG	+	15	6.1	intron (NM_014464, intron 9 of 10)	62735	No
PABPC1	+	15	6.1	Intergenic	-19536	No
AREL1	+	15	6.1	Intergenic	-24769	No
CNTROB	+	15	6.1	Intergenic	-3075	No
none	-	15	6.1	Intergenic	-11181	No

Data show eTF^{SCN1A} binding site sequence hits against the human genome (hg38.p12 assembly); hits showing a homology E-val <10 are reported. Nearest genes are indicated where target sequences were within 100 kb of an annotated gene. Strand indicates genomic strand orientation; length of match indicates the number of contiguous homologous base pairs that are an exact match to the query sequence. Annotation of the target sequence, and distance to TSS are reported from the Homer software package, and any FANTOM5 annotation overlap with target sequence is reported.

Supplementary Table S4. Pre- and post-dose anti-AAV9 neutralizing antibody (NAb) titer in cynomolgus monkey serum and CSF.

Six cynomolgus monkeys with no detectable serum anti-AAV9 NAb were dosed with either AAV9-RE^{GABA}-eTF^{SCN1A} or vehicle control. Serum samples were taken on Day -1 (prior to dosing) and weekly until the end of study (Day 27–29). CSF samples were taken on Day 1 (pre-dose) and Day 24–29, before the end of study. At Day 15, serum levels of anti-AAV9 NAb were not increased in vehicle-treated animals but were increased in AAV9-RE^{GABA}-eTF^{SCN1A}-treated animals, and remained positive at the end of the study with one animal showing further elevated serum titer. CSF samples from pre-dose and Day 24 (vehicle control group) or Day 29 (ETX101-treated group) all tested negative (titer ≤1:5) for anti-AAV9 NAb.

**Test article**	**Dose (vg/animal)**	**Gender**	**Serum**			**CSF**	
			**Day -1**	**Day 15**	**Day 27-Day 29**	**Day 1 (pre-dose)**	**Day 27-Day 29**
Vehicle	NA	M	<1:5	<1:5	<1:5	<1:5	<1:5
		F	<1:5	1:5	<1:5	<1:5	<1:5
AAV9-RE^{GABA}-eTF^{SCN1A} (ETX101a[a])	4.8E13	M	<1:5	1:405	1:405	<1:5	1:5
		F	<1:5	1:405	1:135	<1:5	1:5
AAV9-RE^{GABA}-eTF^{SCN1A} (ETX101s[b])	4.8E13	M	<1:5	1:405	1:1215	<1:5	<1:5
	8.0E13	M	<1:5	1:135	1:135	<1:5	<1:5

[a]ETX101a is produced in adherent HEK293 cells
[b]ETX101s produced in suspension HEK293 cells

Supplementary Table S5. Anti-eTF^{SCN1A} antibody titers in cynomolgus monkey serum and CSF.
(A) Serum samples collected at pre-dose and Day 28 post-dose from 4 AAV9-RE^{GABA}-eTF^{SCN1A}-treated animals were screened for anti-eTF^{SCN1A} antibody at 1:100 sample dilution. Only the post-dose serum from animal #2001 was determined to be anti-eTF^{SCN1A}-positive (OD value above assay cut point). **(B)** The anti-eTF^{SCN1A} antibody titer was determined as 1:400 through serial sample dilution. **(C)** For the animal that tested positive for serum anti-eTF^{SCN1A} antibodies, CSF samples were analyzed for anti-eTF^{SCN1A} antibodies using the same ELISA method. No anti-eTF^{SCN1A} antibodies were detected in the CSF samples taken at Day 28 post-dose with sample dilution as low as 1:5. Data shown are for animal #2001.

(A) Anti-eTF^{SCN1A} antibody screen in cynomolgus monkey serum

Test Article	Dose (vg/animal)	Gender	OD values	
			Day -1	Day 27-Day 29
AAV9-RE^{GABA}-eTF^{SCN1A} (ETX101a[a])	4.8E13	M	0.111	**1.018**[c]
		F	0.103	0.104
AAV9-RE^{GABA}-eTF^{SCN1A} (ETX101s[b])	4.8E13	M	0.143	0.165
	8.0E13	M	0.160	0.140

[a]ETX101a is produced in adherent HEK293 cells
[b]ETX101s produced in suspension HEK293 cells
[c]OD value above floating cut point, indicating anti-eTF^{SCN1A}-positive

(B) Anti-eTF^{SCN1A} antibody titer in serum

Serum sample dilution	Optical density (OD) values	
	Day -1	Day 28
1:100	0.131	**0.734** [a]
1:200	NT	**0.420** [a]
1:400	NT	**0.269** [a]
1:800	NT	0.163

[c]OD value above floating cut point, indicating anti-eTF^{SCN1A}-positive

(C) Anti-eTF^{SCN1A} antibody assay in CSF

CSF sample dilution	Optical density (OD) values	
	Day -1	Day 28
1:5	0.05	0.07
1:10	0.05	0.06
1:20	0.05	0.05
1:40	0.04	0.05
1:80	0.04	0.05
1:160	0.04	0.04
1:320	0.04	0.04
1:640	0.04	0.05
1:1280	0.04	0.05
1:2560	0.05	0.05
1:5120	0.05	0.05
Buffer	0.05	0.05

Supplementary Figure S1. Clustering analysis of CNS cell types in cortical brain tissue.
Heat map showing expression of canonical markers use to annotate cells into one of the following types, as reported in **Fig. 3B**: excitatory neurons (i.e., all cells where CAMK2a is detected); GABAergic inhibitory neurons (i.e. all cells where Gad2 is detected, including cells that have both CAMK2a and Gad2 expression); non-neuronal cells. Cells that expressed a mixed set of markers were annotated as a "Mix" cell type.

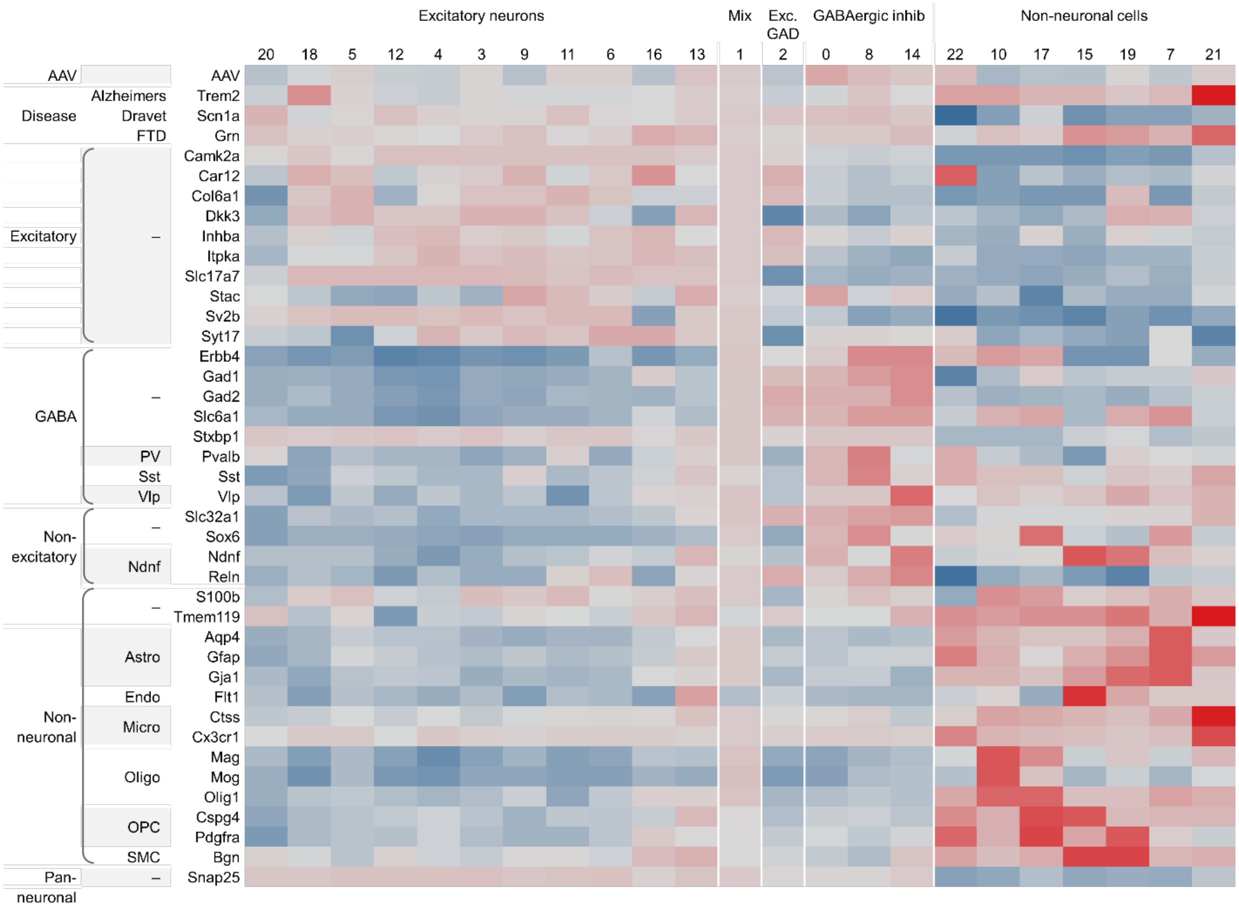

Supplementary Figure S2. Kinetics of vector expression in mouse brain following ICV delivery of AAV9-eGFP.

An AAV9 vector construct identical to the study drug except for the replacement of eTF^{SCN1A} transgene with the reporter gene *EGFP* (AAV9-RE^{GABA}-eGFP) was administered by single bilateral ICV injection to 5 mice at PND1. Representative images are presented, showing GFP expression at different days post injection. Scale bars: 200 µm. DPI, days post injection; GFP, green fluorescent protein; ICV, intra-cerebroventricular; PND, post-natal day.

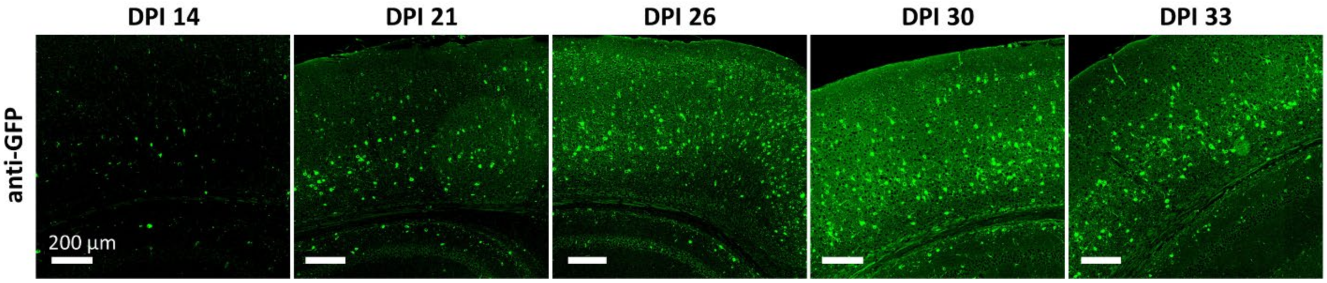

Supplementary Figure S3. Hyperthermia-induced seizures and long-term survival in *Scn1a*$^{+/R1407X}$ Dravet mice.

(A) Bilateral ICV injection of AAV9-RE^{GABA}-eTF^{SCN1A} 5.1E10 vg/animal administered on PND1 protected against febrile seizures; the graph shows the percentage of AAV9-RE^{GABA}-eTF^{SCN1A}-treated *Scn1a*$^{+/-}$ mice experiencing seizures at a given temperature at PND26–PND28; **(B)** AAV9-RE^{GABA}-eTF^{SCN1A}-treated mice had a significantly higher probability of long-term survival compared with controls. **P<0.01 between groups; Log-rank test. ICV, intracerebroventricular; PND, post-natal day; vg, vector genomes.

A

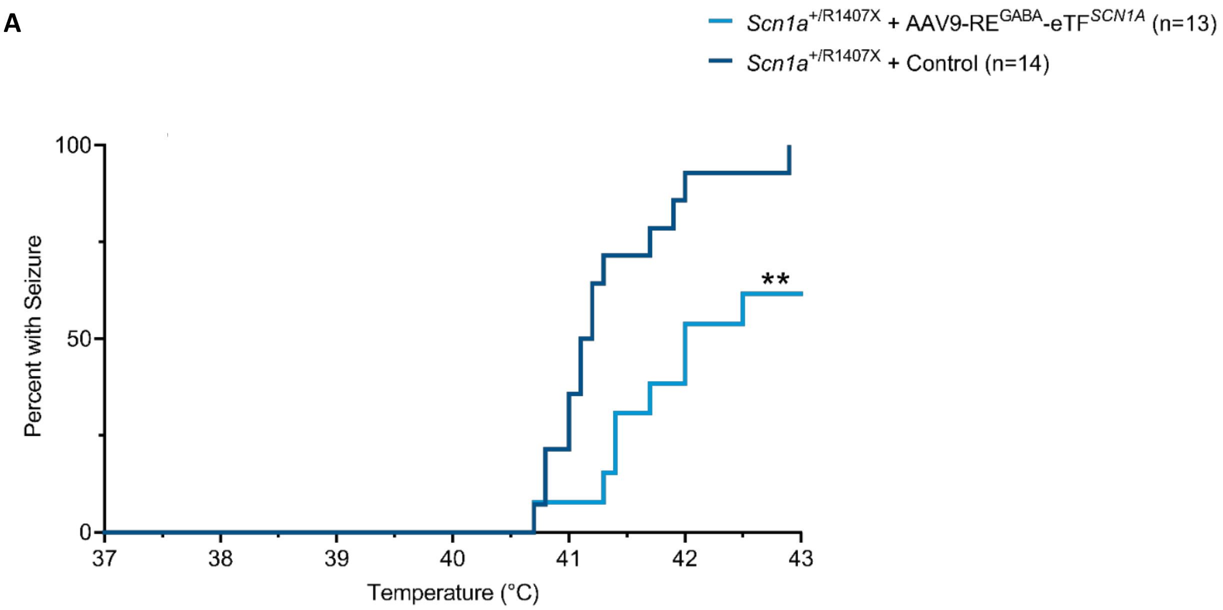

B

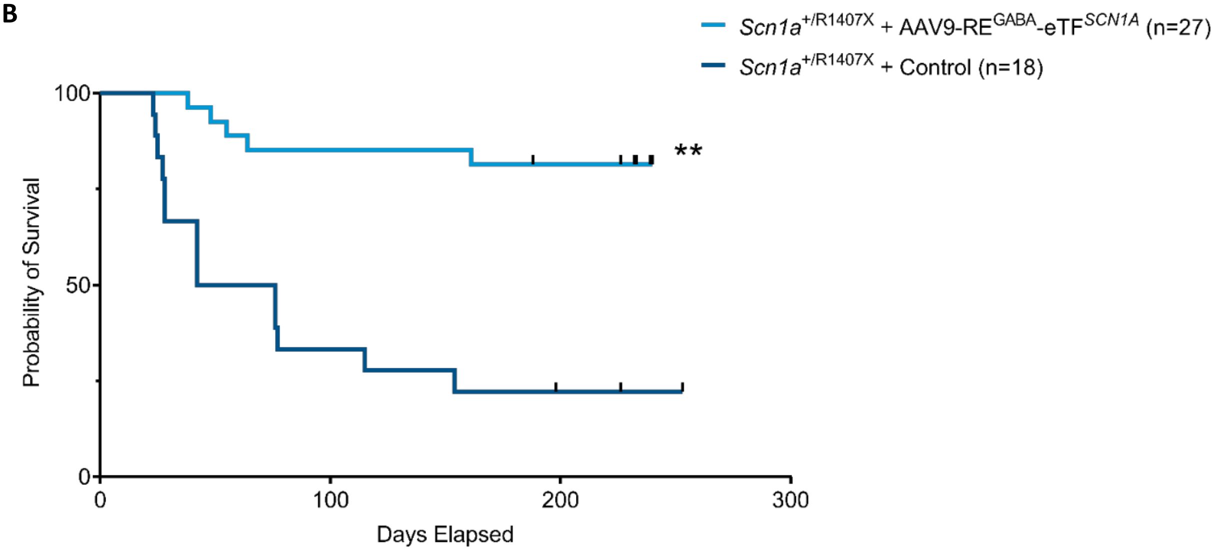

Supplementary Figure S4. Hyperthermia-induced seizures and 90-day survival in *Scn1a*$^{+/-}$ and WT mice dosed at PND5.

Treatment of *Scn1a*$^{+/-}$ mice with AAV9-RE^{GABA}-eTF^{SCN1A} at PND5 prolonged the survival and reduced the mortality rate for up to 90 days as well as reduced the frequency and severity of HTS. **(A) 90-day survival.** WT (n=36 control and n=7 AAV9-RE^{GABA}-eTF^{SCN1A}) and *Scn1a*$^{+/-}$ (n=24 control and n=15 AAV9-RE^{GABA}-eTF^{SCN1A}) mice were dosed at PND5 via bilateral ICV injection (1.34E10 vg/animal; 3 µL/hemisphere). 90-day survival was 87% for *Scn1a*$^{+/-}$ mice treated with vector compared to 12.5% of control-treated animals (p<0.0001, Log-rank test). In contrast, no difference in survival was observed between WT mice treated with AAV9-RE^{GABA}-eTF^{SCN1A} and control-treated animals. WT groups overlap along the 100% line (green and orange lines; only the orange trace is shown). **(B) HTS: percentage of PND5-treated *Scn1a*$^{+/-}$ and WT mice experiencing seizures at a given temperature at PND26–28.** WT (n=14 control and n=10 AAV9-RE^{GABA}-eTF^{SCN1A}) and *Scn1a*$^{+/-}$ (n=9 control and n=10 AAV9-RE^{GABA}-eTF^{SCN1A}) mice were dosed at PND5 via bilateral ICV injection (1.34E10 vg/animal; 3 µl/hemisphere). All of the control-treated *Scn1a*$^{+/-}$ animals exhibited seizures at the maximum temperature of 43.0°C. In contrast, the majority of vector-treated *Scn1a*$^{+/-}$ mice (approximately 78%) remained seizure free at the maximum temperature of 43.0°C (p<0.0001, Log-rank test). WT groups overlap along the zero line (green and orange lines; only the orange trace is shown). HTS, hyperthermia-induced seizure; ICV, intracerebroventricular; PND, post-natal day; vg, vector genomes; WT, wild-type.

A

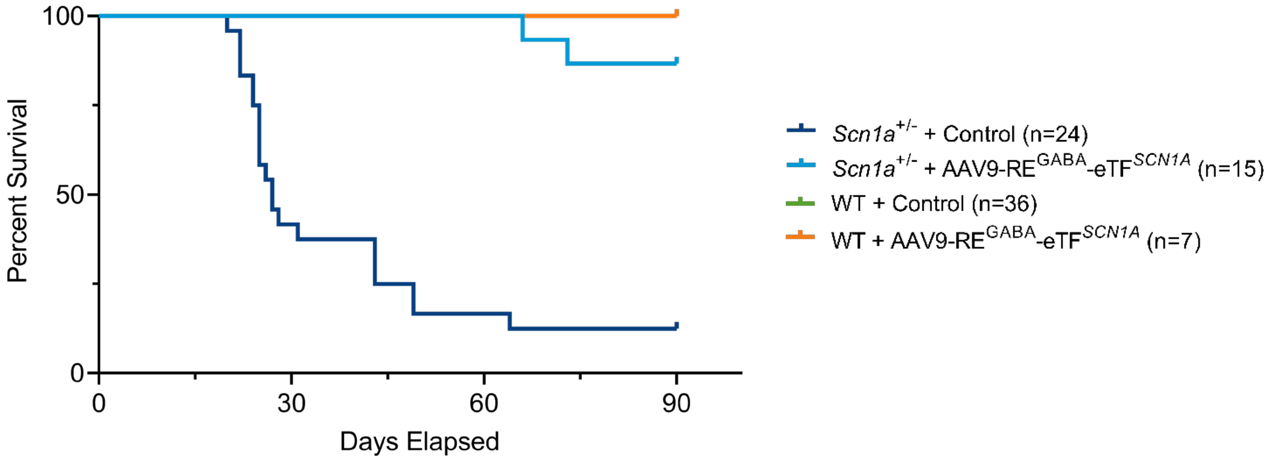

B

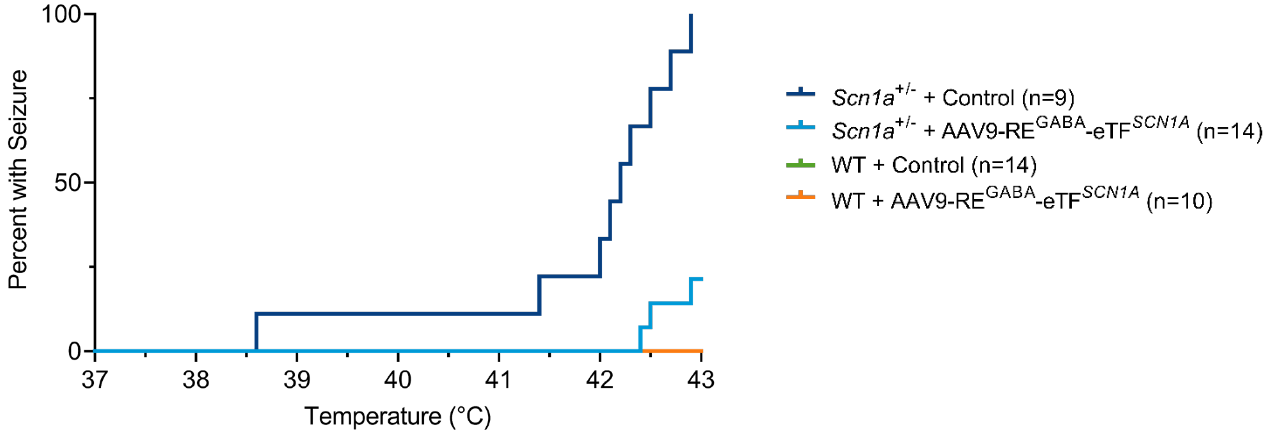

Supplement: Supplemental data [file Suppl_Data.pdf]
